# Strong, Recyclable, Bio‐Based Vitrimers by Tailored Rigid‐Flexible Structures for Advanced Carbon Fiber‐Reinforced Polymers

**DOI:** 10.1002/advs.202513935

**Published:** 2025-10-24

**Authors:** Yong Guo, Nannan Song, Siqi Huo, Cheng Wang, Guofeng Ye, Min Hong, Ye‐Tang Pan, Tingting Chen, Zhongwei Chen, Yuan Yu, Pingan Song, Hao Wang

**Affiliations:** ^1^ Centre for Future Materials University of Southern Queensland Springfield 4300 Australia; ^2^ College of Safety Science and Engineering Nanjing Tech University Nanjing 211816 China; ^3^ School of Engineering University of Southern Queensland Springfield 4300 Australia; ^4^ Hubei Engineering Technology Research Center of Optoelectronic and New Energy Materials School of Materials Science & Engineering Wuhan Institute of Technology Wuhan 430205 China; ^5^ National Engineering Research Center of Flame Retardant Materials School of Materials Science & Engineering Beijing Institute of Technology Beijing 100081 China; ^6^ School of Agriculture and Environmental Science University of Southern Queensland Springfield 4300 Australia

**Keywords:** bio‐based vitrimers, flame retardancy, mechanical performance, recyclable carbon fiber‐reinforced polymers, rigid‐flexible network

## Abstract

Carbon fiber‐reinforced polymers (CFRPs) are typically composed of carbon fibers (CFs) and epoxy (EP) resins, which have been widely utilized in diverse industries. However, the irreversible cross‐linked network of conventional EP resins and their dependence on petroleum‐based resources present serious challenges to the sustainable development of CFRPs. Herein,a bio‐based, high‐performance and recyclable EP (F9T1) is reported by integrating rigid and flexible networks using two fully bio‐based epoxy monomers: i) DGEFA, featuring a rigid conjugated structure, and ii) DGETA, containing a flexible fatty acid backbone with dynamic disulfide groups. Owing to the presence of abundant aromatic structures and disulfide groups, F9T1 features exceptional char‐forming ability, flame retardancy and smoke suppression. Compared with commercial epoxy system (DGEBA), F9T1 shows superior mechanical performance due to its rigid‐flexible network, with enhancements of 56.1%, 19.2% and 28.9% in tensile strength, elongation at break and flexural strength, respectively. The intrinsic degradability of F9T1 enables the fabrication of recyclable CFRPs with improved flame‐retardant and mechanical properties, in which the CFs can be completely reclaimed. Thus, this work establishes a promising design strategy for the creation of next‐generation sustainable thermosetting resins and CFRPs by constructing bio‐based rigid‐flexible dynamic covalent networks.

## Graphical abstract

A sustainable high‐performance epoxy vitrimer (F9T1) was developed via a rigid‐flexible integrated design using rigid and flexible bio‐based epoxy monomers. F9T1 features superior mechanical property, char‐forming ability, fire safety, chemical degradability and recyclability due to its rigid‐flexible network with abundant dynamic disulfide and ester bonds. This makes F9T1 an advanced resin matrix for high‐performance recyclable carbon fiber‐reinforced polymers.

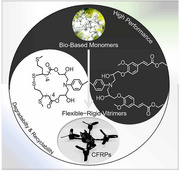

## 1 Introduction

Diglycidyl ether of bisphenol A (DGEBA), as a common epoxy resin (EP), is widely employed as resin matrix in carbon fiber‐reinforced polymers (CFRPs) due to its outstanding mechanical strength, strong adhesion to carbon fibers, and favorable processability.^[^ ^1^ ^]^ However, DGEBA‐based systems face three main challenges: i) environmental concerns stemming from their petrochemical origin and the potential release of bisphenol A (BPA);^[^ ^2^ ^]^ ii) high flammability attributed to the carbon‐hydrogen backbone structure;^[^ ^3^, ^4^ ^]^ and iii) non‐degradability/recyclability due to their permanently cross‐linked network.^[^ ^5^ ^]^ These challenges significantly hinder the sustainable and high‐performance development of EPs.

Driven by the environmental concerns of DGEBA‐based systems, many efforts have been devoted to the development of bio‐based EPs.^[^ ^6^ ^]^ The renewable feedstocks, such as plant oils, lignin, and furfural derivatives, have been explored for the synthesis of bio‐based EPs.^[^ ^7^ ^]^ Notably, some of these bio‐based EP systems have been shown to exhibit comparable mechanical properties and thermal stability to DGEBA‐based system.^[^ ^8^ ^]^ Nonetheless, like DGEBA‐based systems, these bio‐based EP systems are also highly flammable. Introducing flame retardants, such as halogen chemicals and phosphorus derivatives, is a sample yet effective strategy to address the flammability issue of polymeric materials. Despite high efficiency, halogen‐based flame retardants have been progressively phased out due to their adverse environmental and health effects.^[^ ^9^ ^]^ Phosphorus‐derived flame retardants typically enhance flame retardancy at the expense of mechanical properties and chemical resistance. Mainstream flame retardants clearly have significant limitations,^[^ ^10^ ^]^ yet the development of inherently flame‐retardant, halogen/phosphorus‐free, bio‐based EP systems remains highly challenging.

Although great progress has been made in the development of bio‐based EP systems, they still fail to address the environmental issues (e.g., landfill pollution and microplastic contamination) caused by end‐of‐life thermoset products.^[^ ^11^ ^]^ Recent studies have shown that introducing sufficient dynamic covalent bonds into the cross‐linked networks of thermosetting resins can enable the topological rearrangement under external stimuli (e.g., heat and chemical), thereby imparting recyclability and degradability.^[^ ^12^ ^]^ Commonly used dynamic covalent bonds in the fabrication of recyclable thermosets include disulfide,^[^ ^13^ ^]^ Schiff base,^[^ ^14^, ^15^ ^]^ β‐hydroxyl ester,^[^ ^16^, ^17^ ^]^ and siloxane,^[^ ^18^ ^]^ and these thermosets are named vitrimers. While considerable efforts have been devoted to addressing the flammability and non‐recyclability of bio‐based epoxy systems, few have successfully achieved a balanced performance. This is largely due to the inherent conflict between high flame retardancy and mechanical robustness versus degradability and recyclability. Flame‐retardant additives often compromise network integrity or dynamic compatibility, whereas recyclable or degradable networks typically suffer from reduced thermal and mechanical stability. Therefore, designing epoxy systems that reconcile these conflicting requirements remains a critical yet insufficiently explored research challenge.

Herein, we report a novel design strategy for a high‐performance, bio‐based vitrimer (F9T1), achieved by forming a rigid‐flexible dynamic covalent network using two fully bio‐based epoxy monomers (DGETA and DGEFA). This design leverages the rigid conjugated structure of DGEFA to enhance mechanical robustness and char‐forming ability, while the flexible, disulfide‐containing backbone of DGETA provides additional flame‐retardant group and recyclability, enabling a synergistic balance between mechanical robustness, flame retardancy, and sustainability. The resulting F9T1 vitrimer exhibits a high cross‐link density and outstanding durability, with superior tensile strength (88.5 MPa) and flexural strength (137.7 MPa) compared to its counterparts. Furthermore, due to the benzene‐rich structure and disulfide linkages, F9T1 shows excellent flame retardancy, of which the peak heat release rate (pHRR), total heat release (THR), and total smoke production (TSP) are reduced by 45.7%, 54.6%, and 52.5%, respectively, compared to commercial DGEBA. The carbon fiber‐reinforced F9T1 (CF/F9T1) composite demonstrates significantly enhanced performance, of which the carbon fibers can be fully recovered due to the chemical degradability of F9T1. Thus, constructing rigid‐flexible bio‐based dynamic covalent networks can enable the fabrication of high‐performance, sustainable thermosets, combining superior mechanical robustness, flame retardancy and recyclability.

## 2 Results and Discussion

### 2.1 Design of Rigid‐Flexible Integrated Bio‐Based Vitrimer

**Figure 1:**
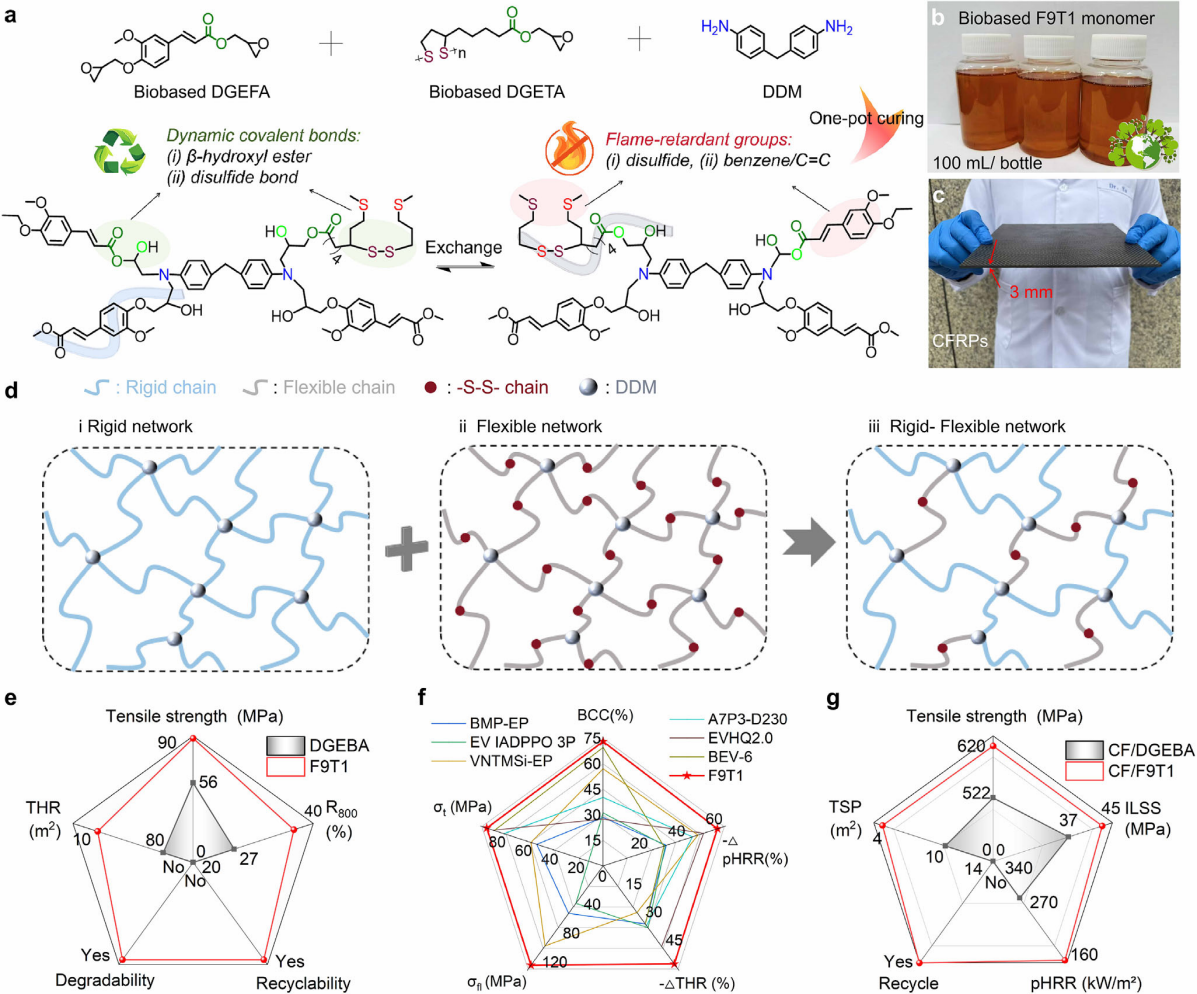
Design of Rigid‐Flexible Integrated Bio‐Based Vitrimer. a) Schematic illustration of the chemical structures for DGEFA, DGETA and DDM and the cross‐linked network of FxTy (x = 9, 8, 7; y = 1, 2, 3); b) Digital photograph of bio‐based F9T1 epoxy monomer prepared on large scale; c) Digital photograph of CF/F9T1 composite; d) Schematic illustrating the formation of the rigid‐flexible network; Overall performance comparison of e) F9T1 and DGEBA, f) F9T1 and previously reported bio‐based flame‐retardant vitrimers^[^ ^19^, ^20^, ^21^, ^22^, ^23^, ^24^ ^]^ and g) CF/F9T1 and CF/DGEBA.

Bio‐based resins have emerged as a promising alternative to petroleum‐based resins, driven significantly by global sustainability initiatives.^[^ ^25^ ^]^ Despite notable progress, current bio‐based thermosets still face challenges in achieving a property portfolio of fire safety, mechanical properties and degradation/recyclability. To simultaneously enhance fire safety and mechanical performance, diverse multifunctional halogen‐ and phosphorus‐based flame retardants have been developed, but they raise significant environmental concerns, such as bioaccumulation and toxic/acidic smoke release during combustion. Herein, high‐performance, halogen/phosphorus‐free, bio‐based (FxTy) thermosets were developed based on a rigid‐flexible integration strategy (**Figure** **1**a; Table S1, Supporting Information). FxTy comprises two bio‐based epoxy resins (DGEFA and DGETA, their mass ratio = x:y) and *4*,*4′*‐diaminodiphenylmethane (DDM, a commercial curing agent). DGEFA is a ferulic acid (FA)‐derived epoxy resin with a rigid conjugated structure consisting of C═C bond and aromatic ring (Scheme S1, Supporting Information), which contributes to fire safety, mechanical robustness and thermal stability (Figure 1d). In contrast, DGETA is a thioctic acid (TA)‐derived epoxy resin with a flexible fatty acid backbone and disulfide groups (Scheme S2, Supporting Information), which imparts toughness and degradation/recyclability (Figure 1d). The structures of DGEFA and DGETA were characterized by Fourier transform infrared (FTIR) spectroscopy, proton nuclear magnetic resonance (^1^H NMR) and Raman spectroscopy (Figure S1, Supporting Information).

FxTy resins are amenable to large‐scale production due to the ease of synthesis for both DGEFA and DGETA, as confirmed by the digital image of F9T1 in Figure 1b. The viscosity of thermosetting resins has a great influence on their processability and industrial applicability.^[^ ^26^ ^]^ Thus, the viscosity of F9T1 resin and F9T1 system was first investigated by a rheometer, with the results shown in Figure S2 (Supporting Information). In Figure S2a (Supporting Information), F9T1 exhibits a viscosity range of 1.2‐1.7 Pa·s between 60 and 160 °C, which is comparable to that of bisphenol A diglycidyl ether (DGEBA, a commercial EP). At 50 °C, F9T1 maintains uncured within 80 min, while DGEBA starts to solidify after 40 min (Figure S2b, Supporting Information), demonstrating a longer processing window for F9T1. In Figure S2c (Supporting Information), DGEBA shows a processing window of 50–143 °C (viscosit < 5 Pa·s), whereas F9T1 exhibits a slightly narrower processing window of 52–122 °C. These findings confirm that both systems feature great processability. The curing procedure (120 °C/2 h, 150 °C/2 h, and 180 °C/2 h) of FxTy was confirmed by differential scanning calorimetry (DSC, Figure S3 and Table S2, Supporting Information). The cross‐linking networks of FxTy were investigated by FTIR, with the results shown in Figure S4a (Supporting Information). After curing, the absorption peak of the epoxy group (913 cm^−1^) disappears in the FTIR spectra, confirming the complete solidification. Meanwhile, the peak of C═C can be found at 1634 cm^−1^, and the reservation of C═C bond contributes to high‐temperature carbonization. The bio‐based carbon contents of FxTy are over 70% (Figure S4b and Table S1, Supporting Information), indicating high bio‐based contents. The thermal stability of FxTy was investigated by thermogravimetric analysis (TGA) with the results presented in Figure S5 and Table S3 (Supporting Information). Notably, F9T1 shows higher char yield at 800 °C (R800, 36.8%) than DGEBA (26.9%), which demonstrates its superior char‐forming ability. Thus, F9T1 features great processibility and superior comprehensive performance, enabling it to be applied as a polymer matrix for carbon fiber‐reinforced polymers (CFRPs), as presented in Figure 1c,e–g.

### 2.2 Excellent Mechanical Robustness and Durability Via Optimized Cross‐linking

**Figure 2:**
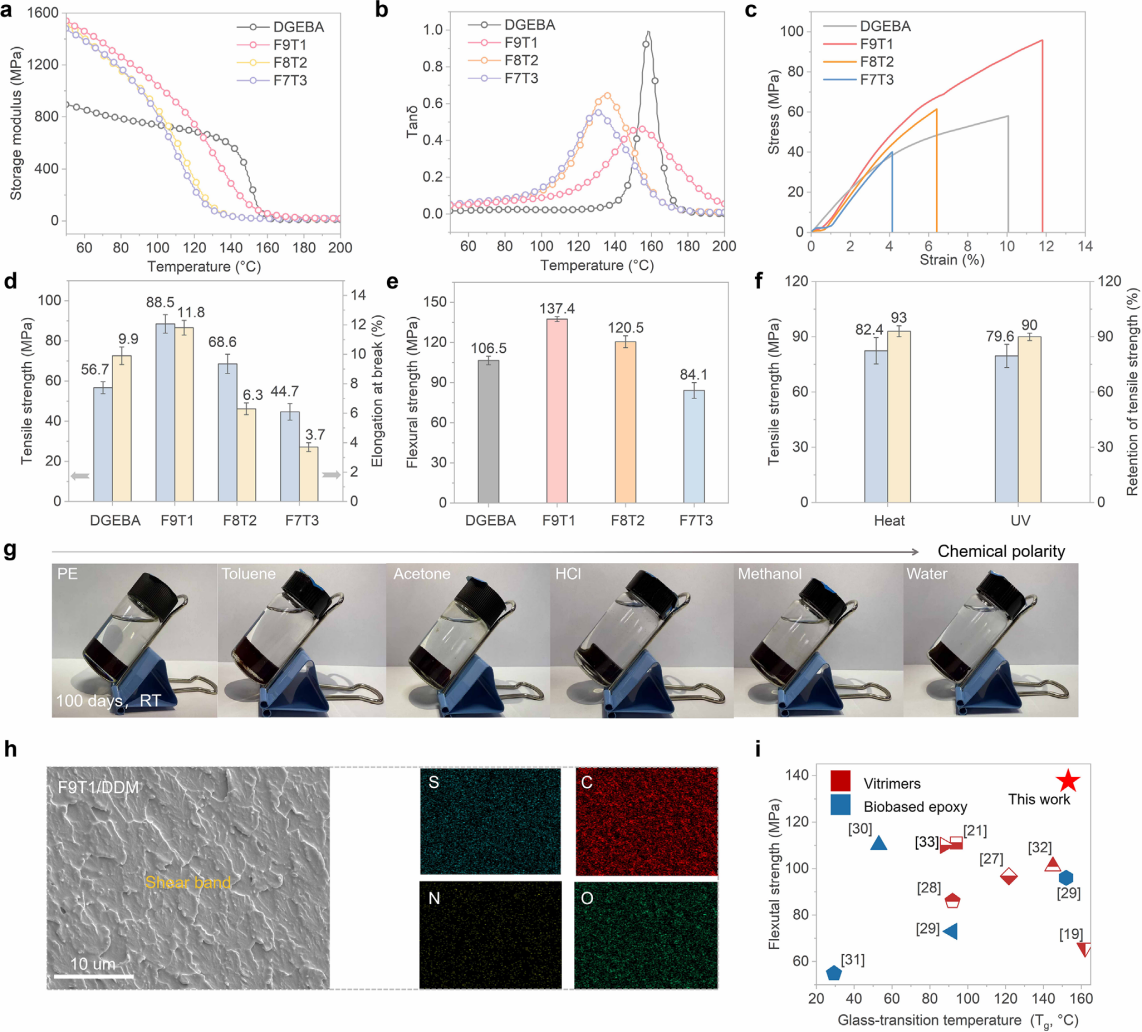
Excellent Mechanical Robustness and Durability via Optimized Cross‐linking. a) Tan δ and b) storage modulus curves of EP samples; c) Tensile stress–strain curves of EP samples; d) Tensile strength, elongation at break, and e) flexural strength of EP samples; f) Tensile strength and tensile strength retention of F9T1 after thermal (100 °C, 7 d) and UV (7 d) aging; g) Digital images of F9T1 soaked in different chemical polarity solvents for 100 d; h) SEM image of the fractured surface of F9T1 with elemental mappings; i) Comparison of flexural strength and glass transition temperature (*T_g_*) between F9T1 and previously reported bio‐based epoxy or vitrimers systems.^[^ ^19^, ^21^, ^27^, ^28^, ^29^, ^30^, ^31^, ^32^, ^33^ ^]^

Dynamic mechanical analysis (DMA) was employed to investigate the viscoelastic behavior of the FxTy networks under thermodynamic conditions, with the results presented in **Figure** **2**a,b and Table S4 (Supporting Information). The storage modulus (E′) of DGEBA at 50 °C is 895 MPa, and its glass transition temperature (*T_g_*) is 158 °C. Owing to the rigid structure in F9T1, it exhibits a significantly higher E′ of 1540 MPa compared to DGEBA, along with a high *T_g_* of 153 °C. According to rubber elasticity theory, the cross‐link density (*V_e_*) of DGEBA is calculated to be 1.00 × 10^3^ MPa. Owing to the rational design of rigid–flexible network structures, F9T1 exhibits a significantly higher *V_e_* of 2.00 × 10^3^ MPa, indicating a denser cross‐linked network and enhanced structural integrity under thermal and mechanical stress. As the DGETA content increases, the dynamic mechanical properties of FxTy are gradually deteriorated, which is probably because of the flexible structure of DGETA.

The mechanical properties of FxTy and DGEBA were investigated in detail, with the results presented in Figure 2c–e and Table S4 (Supporting Information). The tensile strength (σ_t_), elongation at break (δ), tensile toughness (TT) and flexural strength (σ_f_) of DGEBA are 56.7 MPa, 9.6%, 3.82 MJ m^−^^3^ and 106.5 MPa, respectively. Among all FxTy samples, F9T1 demonstrates the best mechanical performance, achieving tensile strength of 88.5 MPa, elongation at break of 11.8%, tensile toughness of 6.74 MJ m^−^^3^ and flexural strength of 137.4 MPa, which are 56.1%, 19.2%, 76.4% and 28.9% higher than those of DGEBA. Similarly, increasing DGETA content compromises the mechanical properties of FxTy. This is mainly due to the long, flexible aliphatic chains in DGETA, which lack strong intermolecular interactions, thus reducing both the tensile strength and toughness of the cured resin network. In addition, F10T0 is rigid but brittle, exhibiting a low impact strength of 2.5 kJ m^−^^2^. When flexible thioctic acid is introduced, the network becomes more balanced; flexible chains help dissipate impact energy while maintaining strength. Notably, F9T1 shows a clear improvement with impact strength increasing to 3.4 kJ m^−^^2^. Notably, the T_g_ and σ_f_ of F9T1 are much higher than those of previously reported bio‐based epoxy or vitrimer systems (Figure 2i; Table S6, Supporting Information), indicative of its superior mechanical robustness and thermal stability.

To evaluate the practical applicability of F9T1, its mechanical stability under harsh environmental conditions, such as UV exposure and high‐temperature, was systematically investigated. The sample was subjected to thermal aging at 100 °C or UV irradiation for 7 d, after which its tensile properties were measured. As shown in Figure 2f and Table S5 (Supporting Information), the tensile strength remains up to 82.4 and 79.6 MPa, respectively, corresponding to retentions of 93% and 90%. Furthermore, F9T1 can retain its structural integrity for over 100 d at room‐temperature when immersed in various polar and non‐polar solvents, including polyethylene (PE), toluene, acetone, hydrochloric acid (HCl), methanol, and water (Figure 2g). These results highlight the exceptional mechanical stability of F9T1, making it a promising candidate for real‐world applications.

The cross‐sectional morphology and element distribution of DGEBA and F9T1 were analyzed by scanning electron microscopy (SEM) and energy‐dispersive X‐ray spectrometry (EDS) to explore the strengthening and toughening mechanism of F9T1. The fracture surface of DGEBA is smooth, indicating a brittle fracture mode (Figure S6, Supporting Information). In contrast, the cross‐section of F9T1 is rough, and numerous shear bands can be observed within the fracture region (Figure 2h). Additionally, the EDS images in Figure 2h reveal the uniform distribution of S, C, N, and O atoms within the fractured surface of F9T1. Mechanistically, the rigid segments of DGEFA and the flexible dynamic chains of DGETA work synergistically to enable effective stress transfer and dissipation, leading to plastic deformation under external forces, enhancing the toughness, mechanical strength, and resistance to crack propagation of F9T1.^[^ ^32^, ^34^ ^]^

### 2.3 Chemically Degradable Vitrimer via Dual Dynamic Bonds

**Figure 3:**
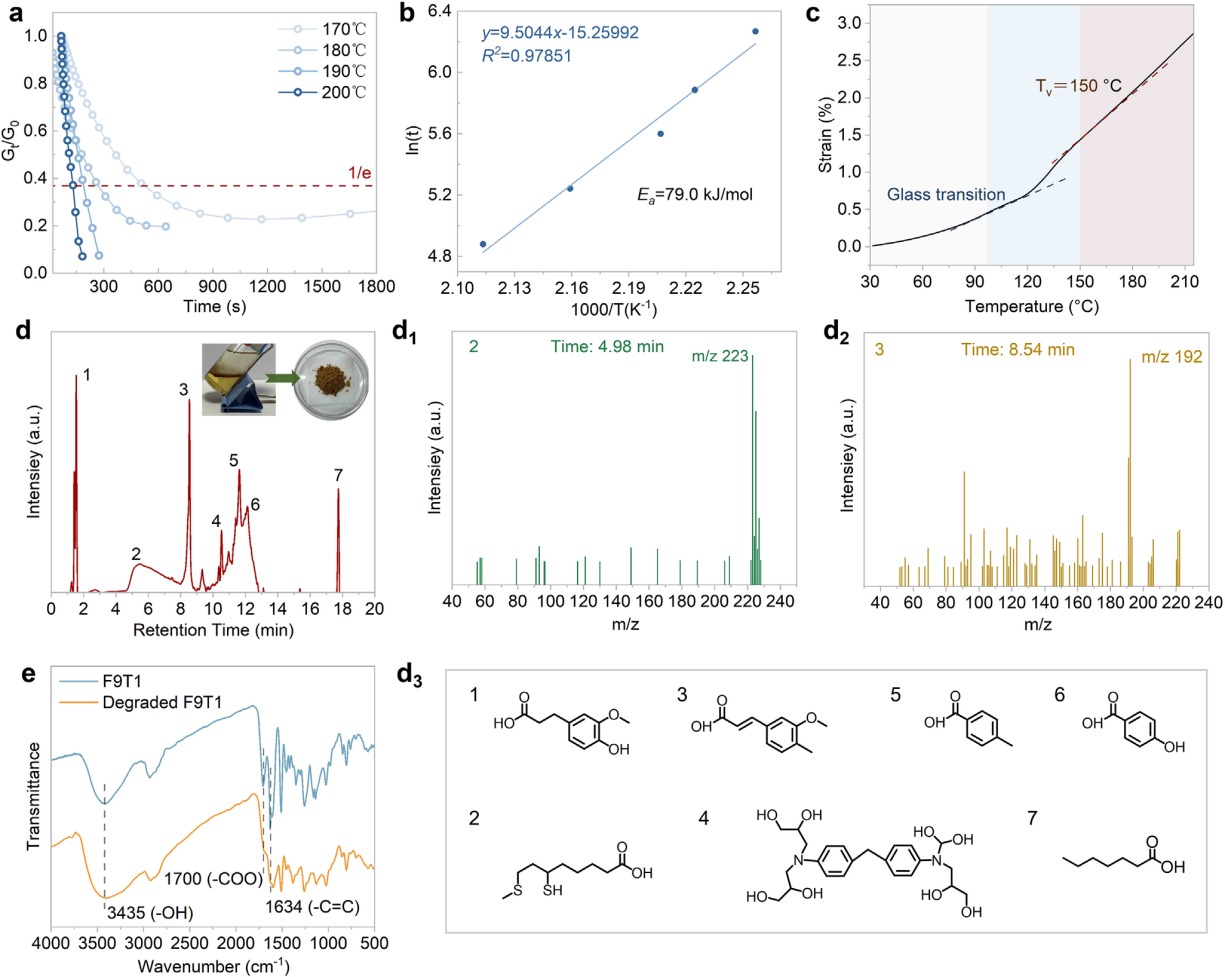
Chemically Degradable Vitrimer via Dual Dynamic Bonds. a) Normalized shear stress relaxation curves of F9T1, and the τ^*^ is indicated by the dashed line (1/e); and b) Arrhenius plot of the measured τ^*^ for F9T1; c) Temperature dependence of the thermal expansion of F9T1; d) HPLC‐MS chromatogram of F9T1 degradation products 1–7 with retention times at 1.53, 4.98, 8.54, 10.5, 11.63, 12.13, and 17.74 min, inset: photograph of F9T1 degraded in THF/NaOH solution (v/v = 8:2) and degradation products after solvent removal; d_1_) Mass spectrum of Peak 2, d_2_) Mass spectrum of Peak 3; d_3_) chemical structures of possible degradation products; e) FTIR spectra of F9T1 and degraded F9T1.

To develop recyclable thermosetting polymers, it is necessary to investigate the topological rearrangement behavior of F9T1 under external stimuli, such as heat and chemicals. The temperature‐dependent stress relaxation of F9T1 was investigated by DMA, where the relaxation time (τ^∗^) was obtained, which was defined as the time required for the initial modulus to decay to 1/𝑒 of its original value. As shown in **Figure** **3**a, the τ^*^ of F9T1 decreases from 525 to 132 s as the temperature increases from 170 to 200 °C, suggesting that topological rearrangement can be facilitated by increasing temperature. A linear fitting of ln(τ^∗^) versus 1000/T (Figure 3b) yields an activation energy (*E*_a_) of 79.0 kJ mol^−1^, which is comparable to previously reported values for EP vitrimers (69–150 kJ mol^−1^).^[^ ^35^ ^]^ The variation of τ^∗^ with temperature follows the Arrhenius equation (Equation 4). The topology freezing transition temperature (*T*_v_) of F9T1 was investigated using the stress‐free expansion method,^[^ ^36^ ^]^ with the curve shown in Figure 3c. The *T*_v_ of F9T1 is ≈150 °C, which further confirms that it can undergo topological rearrangement at elevated temperatures. These results demonstrate that F9T1 is a vitrimer containing dynamic disulfide and ester bonds.

The development of mild degradation conditions for thermosetting polymers facilitates the implementation of sustainable recycling strategies and represents a key pathway to mitigating the increasingly severe environmental issues associated with plastic waste. As shown in Figure S7a (Supporting Information), F10T0 exhibits negligible solubility in THF/NaOH solution (v/v = 8:2) at 90 °C due to its high cross‐linking density and excessive rigid segments. In contrast, F9T1, which incorporates flexible moieties, undergoes efficient degradation under the same conditions. With increasing DGETA content, the FxTy networks become progressively more degradable. As illustrated in Figure S7b,c (Supporting Information), this behavior is primarily attributed to the hydrolysis of disulfide bonds and ester linkages within the FxTy network.^[^ ^37^ ^]^

To gain insights into the degradation mechanism, the chemical structures and molecular weights of the degradation products derived from F9T1 were characterized by high‐performance liquid chromatography–mass spectrometry (HPLC‐MS) and FTIR. As shown in Figure 3d, HPLC‐MS analysis identified seven predominant degradation products (designated as 1–7) with retention times of 1.53, 4.98, 8.54, 10.5, 11.63, 12.13, and 17.74 min, respectively. The corresponding deprotonated molecular ion peaks were clearly observed at m/z 197 [M–H]^−^ (Figure S8a, Supporting Information), m/z 192 [M–H]^−^ (Figure 3d_2_), m/z 134 [M–H]^−^ (Figure S8c, Supporting Information), and m/z 140 [M–H]^−^ (Figure S8d, Supporting Information), which are attributed to degradation fragments derived from the ferulic acid moiety. Peaks at m/z 223 [M–H]^−^ (Figure 3d_1_) and m/z 131 [M–H]^−^ (Figure S8e, Supporting Information) are associated with thioctic acid‐derived degradation products, while m/z 467 [M–H]^−^ (Figure S8b, Supporting Information) corresponds to a fragment originating from the DDM structure. The most plausible chemical structures of these degradation products were proposed and are illustrated in Figure 3d_3_. Furthermore, as shown in Figure 3e, compared to the original F9T1, the degraded F9T1 exhibits a distinct absorption band at 3435 cm^−1^, corresponding to –OH groups, while the carboxyl (–COO^−^) absorption at 1700 cm^−1^ disappears. In addition, the presence of the C═C stretching vibration at 1634 cm^−1^ remains detectable. These spectral changes further support the proposed chemical structures of the degradation products.

### 2.4 Intrinsic, Phosphorus‐Free Flame Retardancy Via Dual‐Mode Action

**Figure 4:**
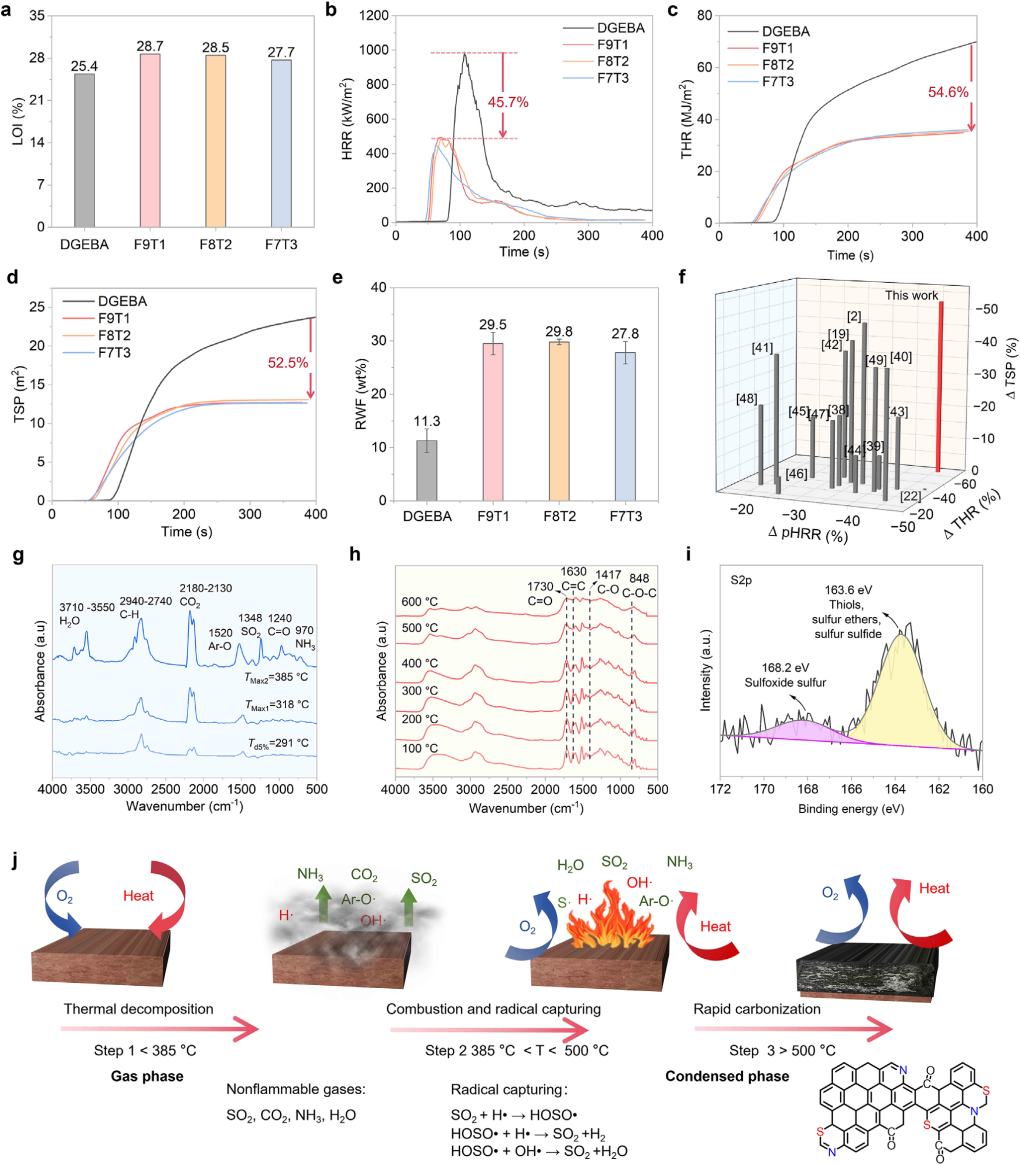
Intrinsic, phosphorus‐free flame retardancy via dual‐mode action. a) LOI values, b) HRR plots, c) THR curves, d) TSP plots, e) RWF values of FxTy samples; f) pHRR, THR, and TSP reductions of the as‐designed F9T1 and previously reported fire‐retardant EP samples.^[^ ^2^, ^19^, ^22^, ^38^, ^39^, ^40^, ^41^, ^42^, ^43^, ^44^, ^45^, ^46^, ^47^, ^48^, ^49^ ^]^ g) FTIR spectra of volatile products during thermal degradation of F9T1; h) In situ FTIR spectra of F9T1 during heating; i) High‐resolution XPS S2p spectrum of F9T1 char residue after CCT; j) Schematic of dual‐phase flame‐retardant behavior of F9T1 during combustion.

The limiting oxygen index (LOI) is a key metric for evaluating the flammability of materials.^[^ ^50^, ^51^, ^52^ ^]^ As shown in **Figure** **4**a and Table S7 (Supporting Information), commercial DGEBA has a relatively low LOI of 25.4%, indicating high flammability. In comparison, FxTy samples have higher LOI values, with the LOI of F9T1 reaching 28.7%, thus they can all be categorized into flame‐retardant materials (LOI > 28%). The LOI of FxTy is gradually reduced with the increasing DGETA content due to the flexible structure of DGETA.^[^ ^53^ ^]^ Cone calorimetry test (CCT) is widely applied to comprehensively investigate the combustion behaviors of materials under forced fire conditions.^[^ ^54^ ^]^ The flame retardancy and smoke suppression of FxTy and DGEBA were investigated by CCT, with the results presented in Figure 4b–e and Table S7 (Supporting Information). DGEBA shows a pHRR of 927.1 kW m^−2^, with a THR of 80.1 MJ m^−2^. In contrast, FxTy exhibits much lower pHRR and THR values. Particularly, the pHRR and THR of F9T1 are decreased to 503.0 kW m^−2^ and 36.4 MJ m^−2^, respectively, with 45.7% and 54.6% reductions relative to those of DGEBA (Figure 4b,c). This confirms that the combustion degree of F9T1 is much lower than that of DGEBA under the same heat flux. Additionally, two critical fire safety parameters, fire performance index (FPI) and fire growth rate (FGR), were calculated, with the results shown in Table S7 (Supporting Information). Generally, higher FPI and lower FGR values indicate improved fire safety and reduced fire hazards.^[^ ^55^, ^56^, ^57^ ^]^ The FPI and FGR values of DGEBA are 0.091 (m^2^ s)/kW and 9.1 kW/(m^2^ s), while those of F9T1 are 0.111 (m^2^ s)/kW and 6.7 kW/(m^2^ s), which further demonstrates the great fire safety for F9T1.

It has been reported that ≈80% of fatalities in fires result from smoke inhalation and toxic gas exposure.^[^ ^58^ ^]^ Therefore, minimizing smoke release during combustion is also important for enhancing fire safety. DGEBA generates a substantial amount of toxic smoke during combustion, with TSP and peak smoke production rate (PSPR) reaching 26.5 m^2^ and 0.265 m^2^ s^−1^, respectively (Figure 4d; Table S7, Supporting Information). In contrast, FxTy consistently demonstrates low smoke production in the burning process. For example, the TSP and PSPR of F9T1 are decreased to 12.6 m^2^ and 0.202 m^2^ s^−1^, which are 52.5% and 23.8% lower than those of DGEBA. F7T3 exhibits 50.2% and 42.6% reductions in TSP and PSPR, relative to those of DGEBA. The remarkable smoke suppression of FxTy is primarily attributed to its superior char‐forming ability.^[^ ^59^ ^]^ In Figure 4e and Table S7 (Supporting Information), the residual weight fraction (RWF) of DGEBA is only 11.3%, whereas those of F9T1, F8T2, and F7T3 reach 29.5%, 29.8%, and 27.8%, respectively. Moreover, the average effective heat of combustion (AEHC) values of FxTy are much lower than that of DGEBA (Table S7, Supporting Information), indicating obviously reduced gas‐phase combustion. Thus, FxTy achieves exceptional flame retardancy and smoke suppression via synergistic condensed‐phase carbonization and gas‐phase flame suppression.

To highlight the fire safety of F9T1, Figure 4f and Table S8 (Supporting Information) compare its pHRR, THR, and TSP reductions with those of previously reported flame‐retardant epoxy systems. The most common strategies for enhancing the flame retardancy of EPs rely on the incorporation of flame retardants, as exemplified by EP/9% U‐DC, EP/2K–NiPS/3DOPO, and EP/DDPS100%. However, these systems show less reductions in pHRR, THR, and TSP compared with F9T1 besides unsatisfactory mechanical properties. Although several halogen/phosphorus‐free epoxy thermosets, such as 15BPPDN/EP and EEU‐DDS, have been developed, they also showed interior flame retardancy and smoke suppression relative to F9T1. Hence, F9T1 stands out for its outstanding combination of flame retardancy and smoke suppression, outperforming both conventional and emerging flame‐retardant EP systems.

Thermogravimetric analysis coupled with infrared spectroscopy (TG‐IR), in situ FTIR, pyrolysis‐gas chromatography/mass spectrometry (Py‐GC/MS), X‐ray photoelectron spectroscopy (XPS), and Raman spectroscopy were applied to investigate the gas‐ and condensed‐phase modes‐of‐action of F9T1. The TG‐IR spectra of the gaseous products for F9T1 and DGEBA under heating are shown in Figure 4g and Figure S9 (Supporting Information). At the initial stage (temperature < 385 °C), the C─O bond breaks first, as confirmed by the reduced intensity of the C─O and C─O─C peaks (1417 and 848 cm^−1^) in the in situ FTIR spectra of F9T1 (Figure 4h), leading to the release of CO_2_. Meanwhile, abundant phenoxy derivatives (1520 cm^−1^) and SO_2_ (1348 cm^−1^) are also released, which can capture and dilute active free radicals to inhibit the gas‐phase combustion.^[^ ^49^ ^]^ Notably, the XPS results (Figure 4i; Table S9, Supporting Information) reveal a low sulfur content in the F9T1 char, further indicating that a significant amount of sulfur is released into the gas phase as SO_2_.^[^ ^60^, ^61^ ^]^ When the temperature rises to 385–500 °C, the absorption peak of NH_3_ emerges at 970 cm^−1^ in the TG‐IR spectra of F9T1, demonstrating the cleavage of C─N bond. Simultaneously, abundant H_2_O molecules are released, as evidenced by the broad absorption band at 3710–3550 cm^−1^. Both NH_3_ and H_2_O function as inert gases, contributing to flame retardancy through dilution effects in the combustion zone. The pyrolysis‐gas chromatography/mass spectrometry (Py‐GC/MS) results in Figures S10 and S11 (Supporting Information) also demonstrate that the sulfur‐ and nitrogen‐containing fragments are released during the pyrolysis of F9T1, and these fragments could be further cracked into SO_2_ and NH_3_. The release of phenoxy derivatives can also be confirmed by Py‐GC/MS. When the temperature exceeds 500 °C, the gas‐phase products are significantly reduced, demonstrating that the matrix is rapidly carbonized at elevated temperatures. Due to the rapid carbonization, significant amounts of nitrogen heterocycle and aromatic structures are remained in the char residue, improving its degree of graphitization and compactness.^[^ ^62^, ^63^ ^]^ Consequently, the structural integrity and degree of graphitization of F9T1 char are much higher than those of DGEBA char, as confirmed by their digital images (Figure S12, Supporting Information) and Raman spectra (Figure S13, Supporting Information). Meanwhile, abundant C═O groups can be detected in the XPS O1s spectrum of F9T1 char (Figure S14, Supporting Information), indicative of its superior oxidation resistance.

As illustrated in Figure 4j, F9T1 undergoes thermal degradation at the early combustion stage, releasing SO_2_, CO_2_ and phenoxy derivatives that can capture and dilute highly reactive H· and OH· radicals (flammable substance).^[^ ^64^ ^]^ As the temperature increases, the inert NH_3_ and H_2_O are released, which also exert diluting effects in the gas phase. Finally, the matrix undergoes rapid carbonization at high temperatures, forming a protective char layer that inhibits combustion in the condensed phase. Specifically, F9T1 integrates rigid conjugated segments from DGEFA with flexible disulfide‐containing segments from DGETA. The existence of abundant conjugated segments enhances the char‐forming ability, while the disulfide groups can degrade into SO_2_ to capture and dilute active free radicals during combustion. Thus, F9T1 features superior flame retardancy and smoke suppression due to the condensed‐phase effect of its conjugated segments and the gas‐phase effect of its disulfide groups.

**Table** **1** provides a comprehensive comparison between F9T1 and previous bio‐based vitrimer systems in terms of bio‐based carbon content, flame retardancy, and mechanical properties. The results clearly highlight the superiority of F9T1, which achieves a high bio‐based carbon content while simultaneously featuring exceptional fire safety and mechanical properties. This balanced performance profile underscores the potential of F9T1 as a promising candidate for sustainable, high‐performance epoxy systems that meet both environmental and safety demands.

**Table 1:** The overall performance comparison between F9T1 and previously reported bio‐based vitrimers.

Reference	Sample	BCC [%]	pHRR reduction [%]	THR reduction [%]	Tensile strength [MPa]	Flexural strength [MPa]
[65]	re‐CP‐co‐BMI	56.3	–	–	7.5	–
[19]	BMP‐EP	28.6	34.0	31.1	50.5	65.9
[66]	E/1C/2D	62.9	54.0	56.2	97.0	–
[67]	FREP10	20.0	23.8	6.4	97.6	131.0
[20]	EV IADPPO 3P	31.2	33.4	33.2	–	52
[68]	EHP0.7T0.7	58.9	54.0	10.6	–	–
[21]	VNTMSi‐DGEBA	57.1	51.0	25.0	54.9	111.0
[22]	A7P3‐D230	40.3	48.0	32.0	75.5	–
[23]	EVHQ2.0	27.6	54.0	43.2	83.5	–
[24]	BEV‐6	69.6	32.7	31.2	82.8	–
This work	F9T1	73.1	45.7	54.6	88.5	137.7

### 2.5 Application in High‐Performance and Recyclable CFRPs

**Figure 5:**
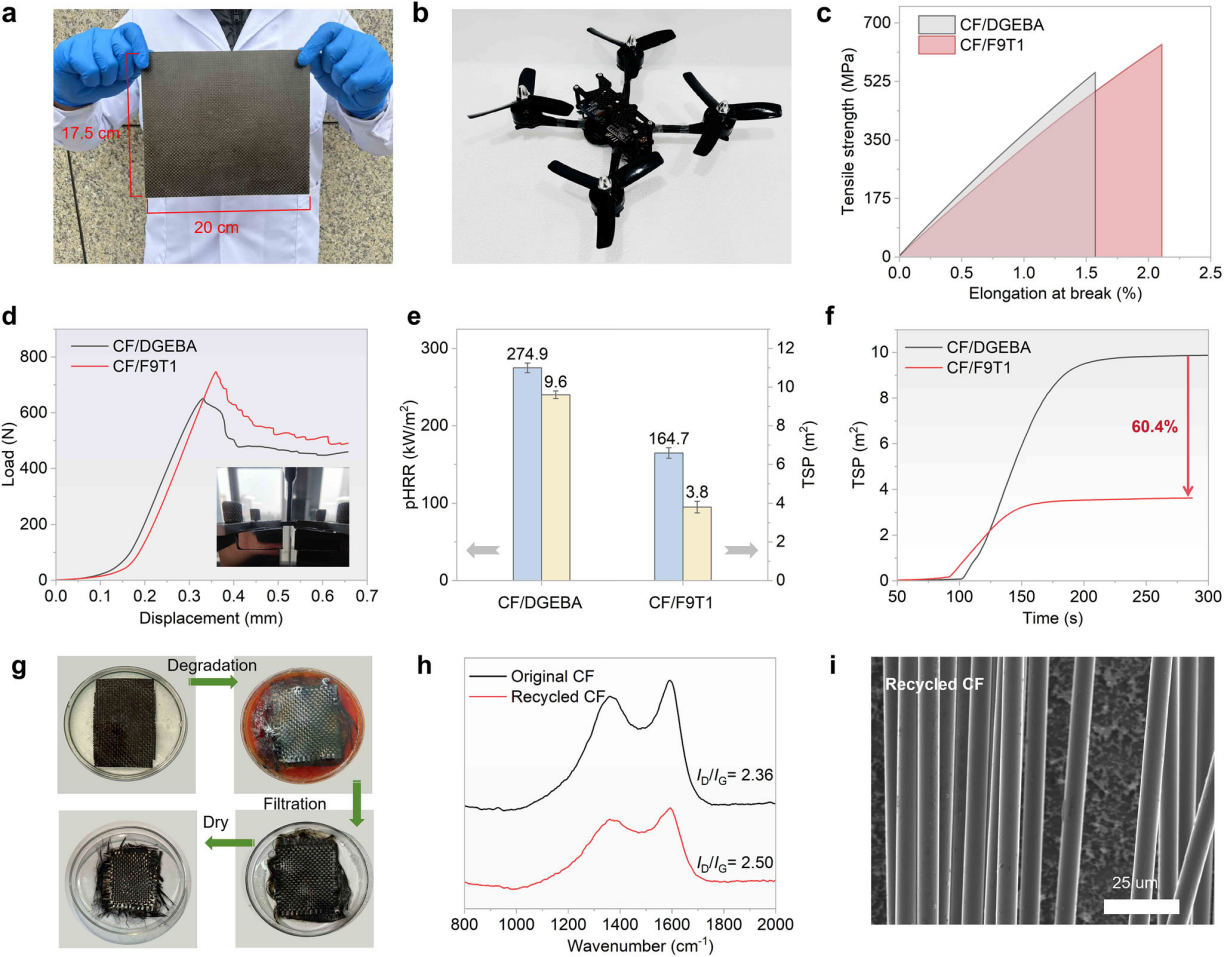
Application in High‐Performance and Recyclable CFRPs. a) Digital photograph of CF/F9T1 composite; b) Digital photograph of unmanned aerial vehicle prepared using CF/F9T1; c) Tensile stress–strain curves of CF/F9T1 and CF/DGEBA; d) Interlaminar shear strength (ILSS) curves of CF/F9T1 and CF/DGEBA; e) pHRR and TSP values of CF/F9T1 and CF/DGEBA; f) TSP curves of CF/F9T1 and CF/DGEBA; g) Illustration of recycling process for CF/F9T1; h) Raman curves of the original and recycled CF; and i) SEM image of the recycled CF.

Due to its high‐performance and vitrimer characteristics, F9T1 can be applied as the resin matrix for recyclable CFRPs (**Figure** **5**a,b). To evaluate the practicality of CF/F9T1, its mechanical properties and flame retardancy were compared with those of commercial carbon fiber‐reinforced DGEBA (CF/DGEBA). Like CF/DGEBA, CF/F9T1 can be prepared by hand lay‐up and hot‐pressing due to their similar processing windows (Figure S2, Supporting Information). In terms of mechanical performance (Figure 5c,d; Table S10, Supporting Information), the tensile strength and interlaminar shear strength (ILSS) of CF/F9T1 reach 621 and 44.7 MPa, which are 19.0% and 20.8% higher than those of CF/DGEBA (522 and 37.1 MPa). As shown in Figure 5e,f and Table S11 (Supporting Information), CF/F9T1 demonstrates significantly enhanced flame retardancy and smoke suppression, with 40.1%, 44.1% and 60.4% reductions in pHRR, THR and TSP, respectively, in comparison to those of CF/DGEBA. These results demonstrate the superior flame retardancy, smoke suppression and mechanical properties of CF/F9T1. Compared with resin matrix, CF is more valuable for recycling because of its higher price and energy consumption. Conventional mechanical, thermal, and chemical recycling methods require harsh conditions (e.g., high‐temperature, pressure, or cutting) and often lead to degradation in the quality or performance of the recycled carbon fibers. Degrading and recycling the resin matrix through special reagents can help to recycle high‐quality carbon fibers in CFRPs, and thus the chemical degradability and recyclability of F9T1 were studied in detail. Hence, given the degradability of F9T1 in THF/NaOH solution at 90 °C, the recyclability of CFs from CF/F9T1 was also investigated under the same conditions. As shown in Figure 5g, by soaking CF/F9T1 in the solution for 6 h, the CFs can be completely recycled. The microstructure and degree of graphitization for the recycled CFs were investigated by SEM and Raman spectroscopy, with the results shown in Figure 5h,i. The recycled CFs display a smooth surface, and their degree of graphitization is close to that of the original CF, confirming that the high‐quality CFs can be recycled in such mild chemical conditions due to the vitrimer nature of F9T1. Furthermore, the F9T1 sample can be regenerated by neutralization, filtration, solvent removal, and hot pressing (Figure S15, Supporting Information). Notably, the reproduced F9T1 sample shows flame retardancy comparable to the original F9T1 sample in CCT (Figure S16 and Table S12, Supporting Information). Hence, the results indicate that F9T1 can be effectively degraded and reused under mild conditions.

These findings underscore the exceptional mechanical performance, fire safety, and recyclability of CF/F9T1, demonstrating its potential in the next generation of lightweight and sustainable composites.^[^ ^69^ ^]^ Due to its superior processability and comprehensive performance, CF/F9T1 has also been successfully applied in the fabrication of structural components for an unmanned aerial vehicle (Figure 5b), demonstrating its practical utility.

## 3 Conclusion 

In summary, we have developed a novel rigid‐flexible network design strategy by molecular engineering for the creation of bio‐based, high‐performance, recyclable thermosets and CFRPs. This strategy combines ferulic acid‐derived epoxy monomers with rigid, benzene‐rich structures and thioctic acid‐derived epoxy monomers with flexible, dynamic backbones to construct a dynamic covalent network that achieves a balance between mechanical robustness, recyclability, and flame retardancy. Specifically, the rigid conjugated segments from DGEFA enhance mechanical robustness and char‐formation capacity, enabling excellent flame retardancy without phosphorus‐based additives. Meanwhile, the flexible, disulfide‐containing segments from DGETA brings about additional flame‐retardant group and improve recyclability. This dynamic covalent network enables efficient, non‐destructive recycling of carbon fibers under mild conditions. Importantly, the integration of rigid conjugated units with flexible dynamic linkages provides a general design principle for tailoring the overall performance of dynamic covalent networks. Thus, this work provides a rational network architecture for developing recyclable, high‐performance, bio‐based thermosets, with potential applicability to polyurethanes, vinyl ester resins, and so on.

## 4 Experimental Section

### Materials

Thioctic acid (TA, ≥ 98%) and sodium chloride (NaCl, 99.6%) were purchased from Shanghai Hanhong Biotechnology Co., Ltd. Epichlorohydrin (ECH, 99%) was obtained from Shanghai Macklin Biochemical Co., Ltd. Triethylbenzyl ammonium chloride (TEBAC, ≥ 98%) was purchased from Shanghai Dibai Biotechnology Co., Ltd. Sodium hydroxide (NaOH, 98%) was acquired from Shanghai Mairui Chemical Technology Co., Ltd. Petroleum ether (PE, 99%) was supplied by Changshu Hongsheng Fine Chemical Co., Ltd. Ferulic acid (FA, 99%), tetrahydrofuran (THF, 99%), and ethyl acetate (EA, 99%) were purchased from Shanghai Jizhi Biochemical Technology Co., Ltd. Methanol (99%) was obtained from Yonghua Chemical Co., Ltd. Acetone (≥ 99.9%), anhydrous magnesium sulfate (MgSO_4_, ≥ 99.0%), and toluene (98%) were provided by Sinopharm Chemical Reagent Co., Ltd. Hydrochloric acid (HCl, 37%) was purchased from Shanghai Linghua Chemical Reagent Co., Ltd. *4*,*4′*‐Diaminodiphenylmethane (DDM, ≥ 97%) was obtained from Aladdin Biochemical Technology Co., Ltd. Epoxy resin used in this study (DGEBA, bisphenol A diglycidyl ether, epoxy equivalent: 210–230 g mol^−1^) was supplied by Nantong Star Synthetic Materials Co., Ltd. Woven carbon fiber fabric (areal density: 200 g m^−2^) was purchased from Yuhui Carbon Fiber Co., Ltd. All chemicals were used as received without further purification.

### Synthesis of Diglycidyl Ether of Ferulic Acid (DGEFA)

FA (36.0 mmol), ECH (720.0 mmol), and TEBAC (3.6 mmol) were placed in a three‐neck flask and stirred at 100 °C for 1 h. A 20% w/w NaOH solution (144.0 mmol) with an additional 3.6 mmol of TEBAC was then added dropwise at 5 °C. The reaction mixture was stirred continuously at 30 °C for 1.5 h. After that, the mixture was extracted using ethyl acetate (33.0 mL) and saturated saline solution (22.0 mL), followed by dehydration with anhydrous MgSO_4_. The final product, diglycidyl ether of ferulic acid (DGEFA, Scheme S1, Supporting Information), was concentrated by rotary evaporation and vacuum‐dried at 80 °C for 12 h, yielding 87%. The epoxy value of DGEFA, determined via hydrochloric acid‐acetone titration, was 0.618 mol/100 g.

**FTIR spectrum (KBr, cm^−1^)**: 1700 (C═O); 1634 (–C═C); 913 (epoxy); 853 (C─O). **^1^H NMR (500 MHz, CDCl_3_)**: δ 7.67 (d, *J* = 15.9 Hz, 1H), 7.13–7.05 (m, 2H), 6.93 (d, *J* = 8.2 Hz, 1H), 6.36 (d, *J* = 15.9 Hz, 1H), 4.56 (dd, *J* = 12.3, 3.0 Hz, 1H), 4.32 (dd, *J* = 11.4, 3.3 Hz, 1H), 4.11–4.01 (m, 2H), 3.93–3.79 (m, 3H), 3.70 (d, *J* = 5.2 Hz, 1H), 3.44–3.37 (m, 1H), 2.95‐2.87 (m, 2H), 2.83‐2.75 (m, 1H), 2.74‐2.67 (m, 1H).

### Synthesis of Disulfide‐Based Epoxy Diluent (DGETA)

TA (36.0 mmol), ECH (720.0 mmol), and TEBAC (3.6 mmol) were introduced into a three‐neck flask and stirred at 100 °C for 1 h. A 20% w/w NaOH solution (144.0 mmol), along with an additional 3.6 mmol of TEBAC, was then added dropwise to the mixture at 5 °C, followed by stirring at 30 °C for 1.5 h. The resulting mixture was extracted using ethyl acetate (33.0 mL) and a saturated saline solution (22.0 mL), followed by dehydration with anhydrous MgSO_4_. Finally, the product (DGETA, Scheme S2, Supporting Information) was concentrated using a rotary evaporator and vacuum‐dried at 80 °C for 12 h, yielding 40%.

**FTIR spectrum (KBr, cm^−1^)**: 1740 (C═O); 913 (epoxy); 853 (C─O). **^1^H NMR (600 MHz, CDCl_3_)**: δ 4.40 (dd, *J* = 12.3, 3.0 Hz, 1H), 3.97–3.85 (m, 1H), 3.58‐3.51 (m, 1H), 2.91–2.55 (m, 6H), 2.36 (t, *J* = 7.4 Hz, 2H), 1.94–1.81 (m, 1H), 1.71–1.56 (m, 4H), 1.51‐1.29 (m, 2H) ppm. **Raman Spectrum (λ = 633 nm, cm^−1^)**: 506 and 520 (polymerized S‐S); 675 (C‐S).

### Preparation of EP Vitrimers and Carbon Fiber Reinforced Composites

The EP vitrimers were manufactured by the curing reaction between DGEFA and DGETA with DDM. The molar ratio of N‐H groups in DDM to epoxy groups in DGEFA and DGETA was maintained at 1:1, and the curing procedure was 120, 150, and 180 °C for 2 h, respectively. The formulas of EP samples are listed in Table S1 (Supporting Information). The DDM‐cured DGEFA/DGETA‐containing resin was denoted as FxTy, where x and y represent the contents of DGEFA and DGETA, respectively. The DDM‐cured DGEBA was denoted as DGEBA, as a control group. According to ISO 16620–2:2019, the bio‐based content is defined as the percentage of carbon atoms in a product that are derived from renewable biomass sources, relative to the total carbon content of the product. The bio‐based content can be calculated by the following Equation 1:

(1) Bio−basedcontent(%)=(Ctotal/Cbio)×100%

Where:

*C*_bio_ = amount of carbon derived from biomass in the product, *C*_total_ = total carbon content in the product

The carbon fiber‐reinforced F9T1 and DGEBA (CF/F9T1 and CF/DGEBA) composites were prepared by hand lay‐up, followed by hot‐pressing at 120, 150, and 180 °C for 2 h, respectively, under a pressure of 18 MPa. The weight fraction of CF cloth in the composites was ≈60 wt.%.

## 5 Characterizations

### 5.1 Structure Analysis

Fourier transform infrared (FTIR) spectra were obtained using a Nicolet IS5 spectrometer (Thermo Fisher Scientific, USA). Proton nuclear magnetic resonance (^1^H NMR) spectra were recorded on a Bruker AV400 spectrometer (Bruker, Switzerland). Raman spectra were collected at room‐temperature using a LabRAM HR Evolution Raman spectrometer (HORIBA, France) with a 633 nm argon laser as the excitation source.

### 5.2 Curing Behaviors, Rheological Properties, and Thermal Analysis

Curing behaviors were investigated using a TA DSC 25 differential scanning calorimeter (TA Instruments, USA). The samples were heated from 40 to 200 °C at heating rates of 5, 10, 15, and 20 °C min^−1^ to analyze the curing kinetics. The activation energy (*E*□) was calculated according to Kissinger's method:

(2) $$\ln\left(\frac{\beta }{T_{p}^{2}}\right)=\ln\left(\frac{AR}{E_{a}}\right)\;-\frac{E_{a}}{RT_{p}}$$

where *β*, *T*□, *A*, and *R* represent the heating rate, peak temperature, pre‐exponential factor, and gas constant (8.314 J·mol^−1^·K^−1^), respectively.

The rheological properties of epoxy resins were measured using an MCR302 rheometer (Anton Paar, Austria). The dynamic rheological behaviors of the epoxy systems were tested in oscillatory mode with a heating rate of 5 °C min^−1^, a test frequency of 1 Hz, and a constant stress of 10 Pa. Thermogravimetric analysis (TGA) was performed using a TGA/DSC 3+ thermal analyzer (Mettler Toledo, Switzerland) under nitrogen atmosphere at a heating rate of 20 °C min^−1^ from 50 to 800 °C.

### 5.3 Mechanical Measurements and Durability Evaluation

Dynamic mechanical analysis (DMA) was carried out on a dynamic mechanical analyzer (MCR 302, Anton Paar, Austria). Rectangular specimens (50 mm × 10 mm × 1 mm) were tested from 50 to 200 °C at a heating rate of 3 °C min^−1^ and a frequency of 1 Hz. The cross‐link density (*V*_e_) was calculated using the storage modulus (*E’*) in the rubbery plateau region according to the following equation:

(3) $$v_{e}=\frac{E^{\prime }}{3RT}\;$$

where *E’* is the storage modulus at a temperature 30 K above the glass transition temperature (*T*_g_), *R* is the gas constant, and *T* is the absolute temperature.

Flexural and tensile properties of EP samples were measured at room‐temperature using a WDW‐20 universal testing machine (Changchun Xinte Testing Machine Co., China) in accordance with ISO 178 and ISO 527 standards. The reported values are the average of five specimens. Tensile toughness was calculated from tensile stress–strain curves in Figure 2c. Impact properties of EP samples were measured at room‐temperature using a XC‐22D cantilever beam impact testing machine(Chengde Precision Testing Machine Co., Ltd, China) in accordance with ISO 180. Elemental mapping was performed using an Oxford X‐MAX energy‐dispersive X‐ray spectrometer (EDS) operated at an accelerating voltage of 5 kV. Thermal aging was performed in an oven at 100 °C for 7 d. Ultraviolet (UV) aging tests were carried out using a UV accelerated weathering tester (UVA/se, Q‐Lab, USA). The samples were exposed to UVA light at a wavelength of 340 nm, with an irradiance of 0.51 W m^−2^ and a black panel temperature of 65 °C for 7 d. After aging, the tensile strength of the obtained samples was measured. Tensile tests of carbon fiber reinforced composites were conducted using a Zwick/Roell Z1.0 universal testing machine (Germany), based on ASTM D3039 standard. The specimen dimensions were 220 mm × 15 mm × 1 mm. Interlaminar shear strength (ILSS) of carbon fiber reinforced composites was evaluated using a CMT4304 electronic universal testing machine (China), based on ASTM D2344 standard. The test was carried out on specimens with sizes of 15 mm × 4 mm × 2 mm, and the span length and loading rate were 8 mm (four times the thickness) and 1 mm min^−1^, respectively.

### 5.4 Viscoelastic Relaxation, Topological Transition tests and Degradation Behavior

Stress relaxation behavior was characterized by using a Q800 dynamic mechanical analyzer (DMA, TA Instruments, USA) in tension mode. Specimens with dimensions of 30 mm × 6 mm × 1 mm were tested at a frequency of 1 Hz. A preload of 1 × 10^−3^ N was applied for 10 min at the target temperature, followed by a 1% strain. The decay of the relaxation modulus was monitored, and the relaxation time (τ^*^) was defined as the time when the modulus (G_t_) decreased to 1/e of the original value (G_0_). The temperature dependence of τ^*^ followed the Arrhenius equation:

(4) $$\ln\tau^{*}=\;\ln\tau_{0}+E_{a}/RT$$

where τ_0_ is the characteristic relaxation time at infinite temperature, *E*_a_ is the activation energy, *R* is the gas constant, and *T* is the absolute temperature.

The topology freezing temperature (*T*_v_) was determined using the thermal expansion method on a Q800 dynamic mechanical analyzer (DMA, TA Instruments, USA). A constant stress of 2 kPa was applied to samples (dimensions: 30 mm × 6 mm ×1 mm), and the strain evolution was recorded as the temperature increased from 30 to 220 °C at a rate of 10 °C min^−1^.

HPLC‐MS analysis was conducted using a Thermo Scientific Q Exactive with an Ultimate 3000 UPLC system. Samples were separated on an XBridge Premier BEH C18 column (2.1 × 100 mm, 2.5 µm) using a gradient of 0.1% formic acid in acetonitrile and water at 0.2 mL min^−1^. Mass spectra were recorded in ESI± mode with a spray voltage of 3200 V and a capillary temperature of 300 °C.

### 5.5 Fire Testing and Fire‐Retardant Mechanism

The limiting oxygen index (LOI) was measured using a JF‐3 oxygen index tester (Jiangning Analytical Instrument Co., China) based on ASTM D2863 standard. The dimensions of specimens were 120 mm × 10 mm × 4 mm. Cone calorimeter testing (CCT) was conducted using an iCone mini instrument (Fire Testing Technology Ltd., UK) to evaluate the combustion behavior of EP samples and their composites. The tests were carried out on sheets with sizes of 100 mm × 100 mm × 5 mm under an external flux of 35 kW m^−2^, in accordance with ISO 5660–2. The corresponding flame‐retardant index was calculated based on the acquired data:

(5) Fireperformanceindex(FPI)=TTI/pHRR

(6) Firegrowthrate(FGR)=pHRR/TpHRR

Pyrolysis‐gas chromatography/mass spectrometry (Py‐GC/MS) analysis was carried out using a GCMS‐QP2010 Ultra system equipped with an EGA/PY3030D pyrolyzer (Shimadzu, Japan). The pyrolysis was conducted in a helium atmosphere at 500 °C with a heating rate of 1000 °C min^−1^ for 20 s. Thermogravimetric analysis coupled with infrared spectroscopy (TG‐IR) was performed using a TGA2 thermal analyzer (Mettler Toledo, Switzerland) connected to a TENSOR II FTIR spectrometer (Bruker, Germany). The measurements were conducted under nitrogen with a heating rate of 20 °C min^−1^ from 40 to 850 °C. X‐ray photoelectron spectroscopy (XPS) was conducted on an ESCALAB XI+ spectrometer (Thermo Fisher Scientific, USA) using Al Kα radiation. Scanning electron microscopy (SEM) images were acquired using a Quanta 400 FEG microscope (Thermo Fisher Scientific, USA). In situ FTIR was performed using a Nicolet iS50 spectrometer (Thermo Fisher Scientific, USA) under nitrogen atmosphere, with a heating rate of 10 °C min^−1^ from 50 to 600 °C.

### 5.6 Characterization of Recycled Carbon Fiber

The microstructure and degree of graphitization for the recycled carbon fiber were investigated by SEM and Raman spectroscopy, and the testing conditions were as described above.

## Conflict of Interest

The authors declare no conflict of interest.

## Supporting information



Supporting Information

## Data Availability

Data sharing is not applicable to this article as no new data were created or analyzed in this study.
