# Supplementary material for: Strong, Recyclable, Bio‐Based Vitrimers by Tailored Rigid‐Flexible Structures for Advanced Carbon Fiber‐Reinforced Polymers
Source: Adv Sci (Weinh). 2025 Oct 24;13(3):e13935. doi: 10.1002/advs.202513935 (PMC12806494; doi:10.1002/advs.202513935)
Supplement: Supplementary file 1 — Supporting Information [file ADVS-13-e13935-s001.docx]

**Supporting Information**

**Strong, Recyclable, Bio-Based Vitrimers by Tailored Rigid-Flexible Structures for Advanced Carbon Fiber-Reinforced Polymers**

*Yong Guo,* *Nannan Song, Siqi Huo*, Cheng Wang, Guofeng Ye, Min Hong, Ye-Tang Pan, Tingting Chen, Zhongwei Chen, Yuan Yu, Pingan Song*, Hao Wang**

Y. Guo, S. Huo, M. Hong, P. Song, H. Wang

Centre for Future Materials, University of Southern Queensland, Springfield 4300, Australia

E-mail: Siqi.Huo@unisq.edu.au, sqhuo@hotmail.com (S. Huo)

E-mail: pingansong@gmail.com, pingan.song@usq.edu.au (P. Song)

E-mail: hao.wang@unisq.edu.au (H. Wang)

S. Huo, M. Hong, H. Wang

School of Engineering, University of Southern Queensland, Springfield 4300, Australia

N. Song, T. Chen, Z. Chen, Y. Yu

College of Safety Science and Engineering, Nanjing Tech University, Nanjing 211816, China

C. Wang, G. Ye

Hubei Engineering Technology Research Center of Optoelectronic and New Energy Materials, School of Materials Science & Engineering, Wuhan Institute of Technology, Wuhan 430205, China

Y.-T. Pan

National Engineering Research Center of Flame Retardant Materials, School of Materials Science & Engineering, Beijing Institute of Technology, Beijing 100081, China

P. Song

School of Agriculture and Environmental Science, University of Southern Queensland, Springfield 4300, Australia

**This supporting information includes:**

Characterization of DGEFA and DGETA

Supplementary Figure S1-S17

Supplementary Table S1-S12

1. Characterization of DGEFA and DGETA

The chemical structure of DGEFA and DGETA was characterized *via* FTIR, NMR, and Raman, with the results presented in Figure S1. As shown in Figure S1a, the -OH group of FA, initially observed at 3435 cm^-1^, disappears in the FTIR spectrum of DGEFA, and a characteristic peak of epoxy appears at 913 cm^-1^. Additionally, distinct absorption peaks corresponding to the C=O and C=C bonds are observed at 1740 and 1634 cm^-1^, respectively, in the FTIR spectrum of DGEFA. The epoxy peak can also be detected at 913 cm^-1^ in the FTIR spectrum of DGETA, and the peaks at 853 and 1740 cm^-1^ are attributed to C-O and C=O bonds, respectively. The ^1^H NMR of DGEFA (Figure S1b) display obvious proton signals in the range of 3.70-2.67 ppm, corresponding to the epoxy groups. Similarly, the ^1^H NMR of DGETA (Figure S1c) exhibits signals at 4.40-3.51 ppm and 2.9-2.55 ppm, also attributed to epoxy functionalities. The retention of other structural signals confirms the successful completion of both epoxidation reactions. Furthermore, in the Raman spectrum of DGETA (Figure S1d), the peaks at 506 and 520 cm^-1^ belong to the polymerized -S-S- bonds, while the peak at 675 cm^-1^ is assigned to C-S stretching vibrations. These results verify the successful synthesis of both epoxy monomers, DGEFA and DGETA.


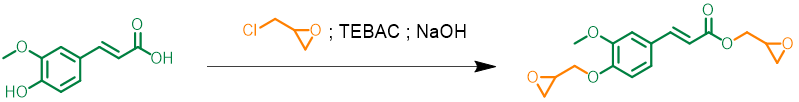
**Scheme S1.** The synthetic route of DGEFA.


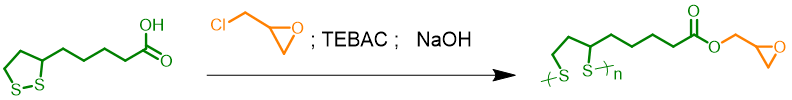


**Scheme S2.** The synthetic route of DGETA.


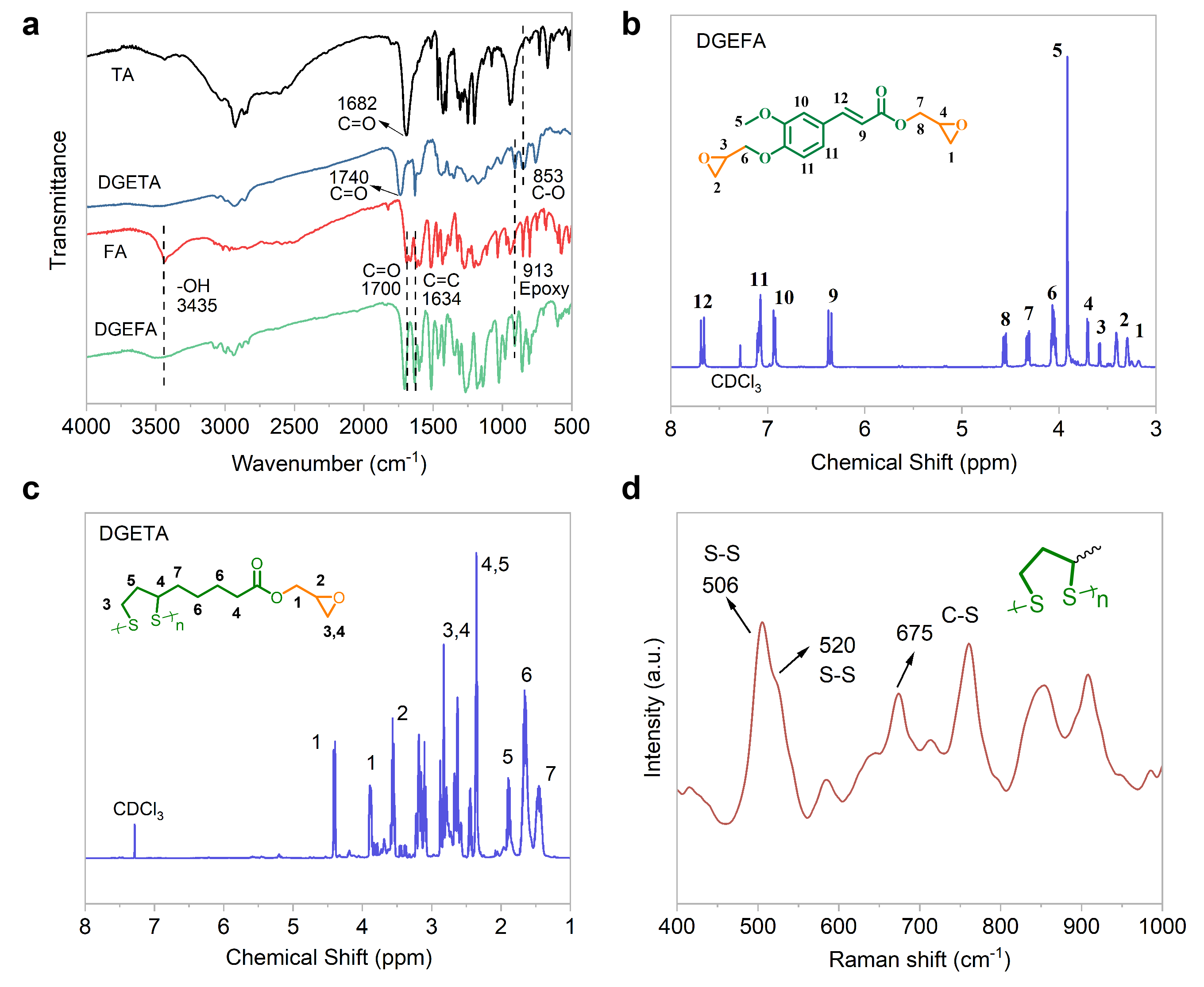


**Figure S1.** (a) FTIR spectra of TA, DGETA, FA and DGEFA; (b) ^1^H NMR spectrum of DGEFA; (c) ^1^H NMR spectrum of DGETA; and (d) Raman spectrum of DGETA.


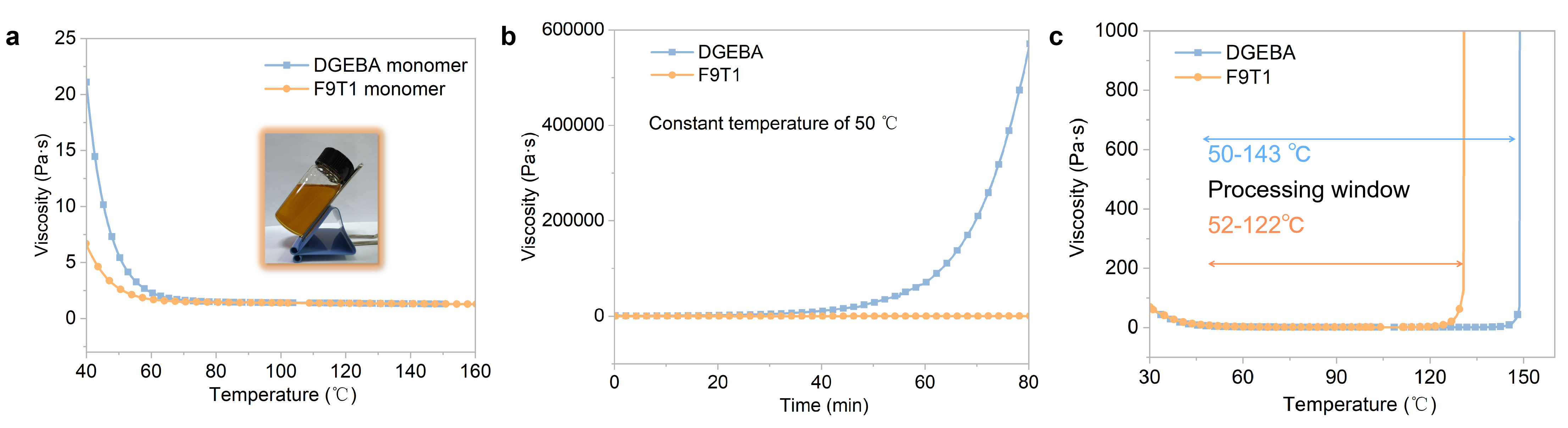


**Figure S2.** (a) Viscosity-temperature curves of DGEBA monomer and F9T1 monomer, with a photograph of liquid F9T1 monomer; (b) Viscosity-time curves of DGEBA and F9T1 at 50 °C; and (c) Viscosity-temperature curves of uncured DGEBA and F9T1 from 30-150 °C, with their processing window.


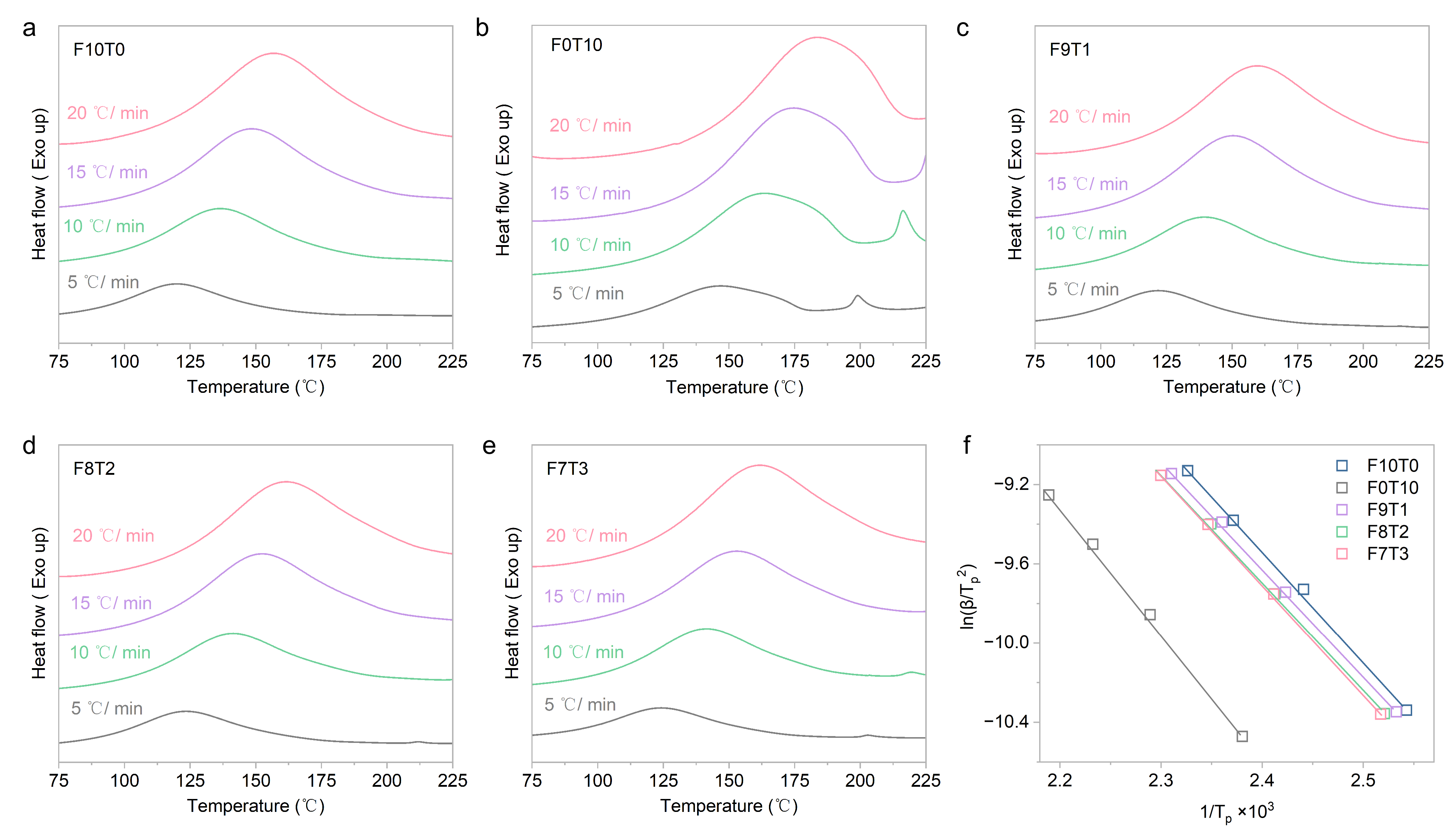


**Figure S3.** Non-isothermal DSC curves of (a) F10T0, (b) F0T10, (c) F9T1, (d) F8T2 and (e) F7T3 at different heating rates; and (f) Linear pots of ln(β/*T*_p_^2^) versus 1/*T*_p_×10^3^ for different epoxy systems based on Kissinger’s equation.


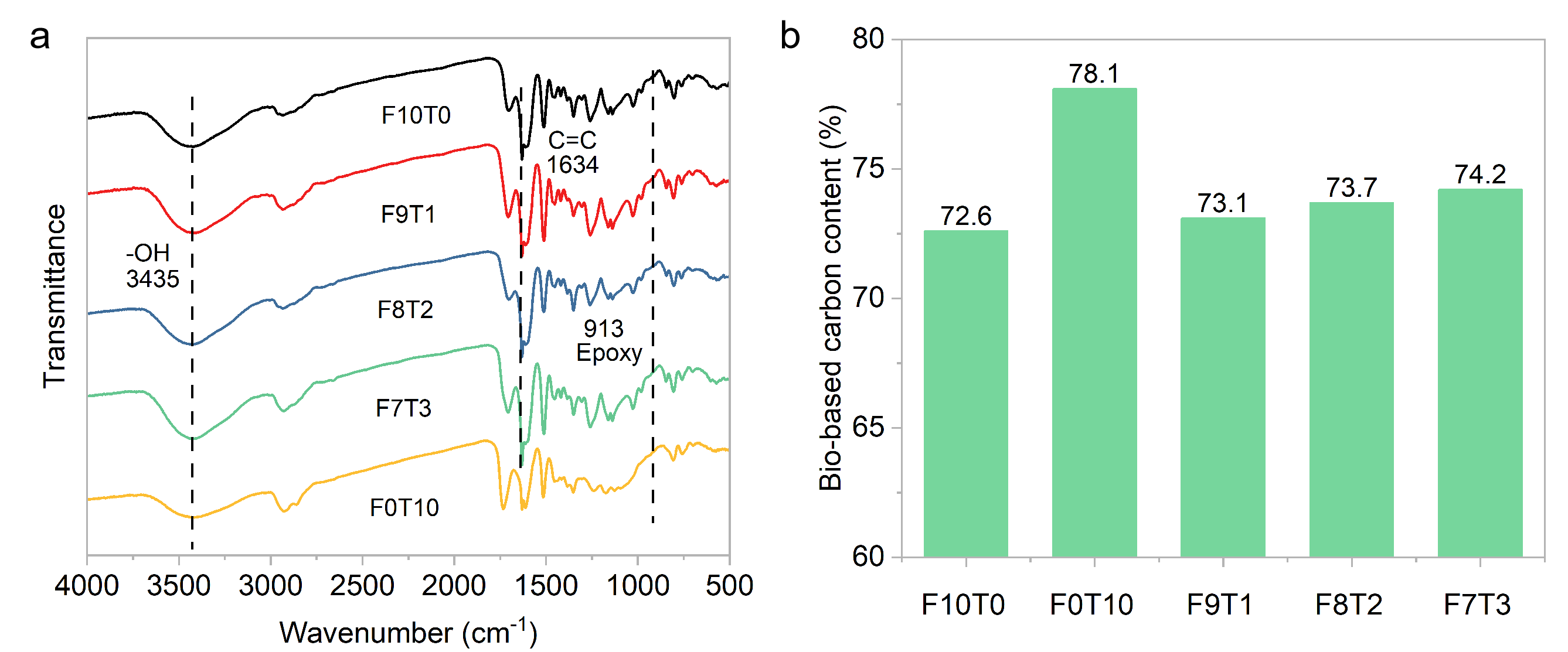


**Figure S4.** (a) FTIR spectra of different epoxy thermosets; and (b) Bio-based carbon content of FxTy/DDM (x = 0,10,9, 8, 7; y = 0,1, 2, 3,10).


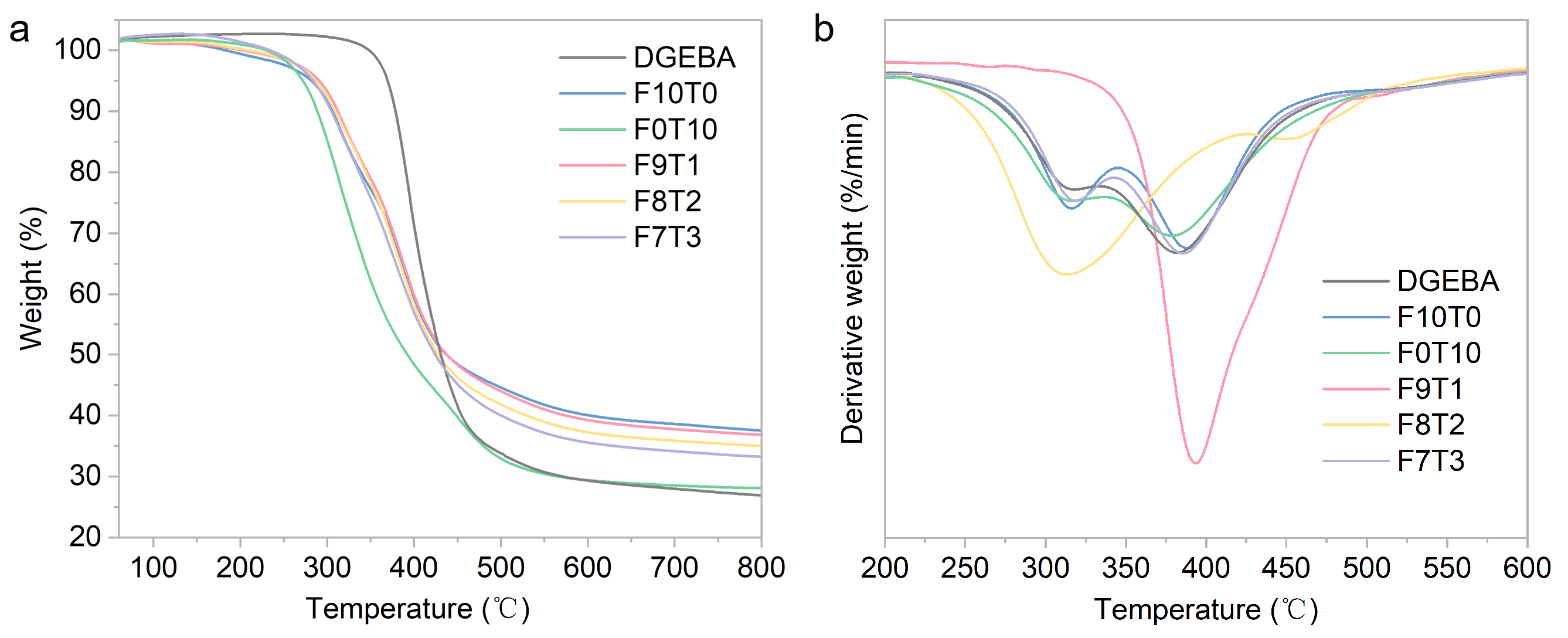


**Figure S5.** (a) TG and (b) DTG curves of EP samples in N_2_ condition.


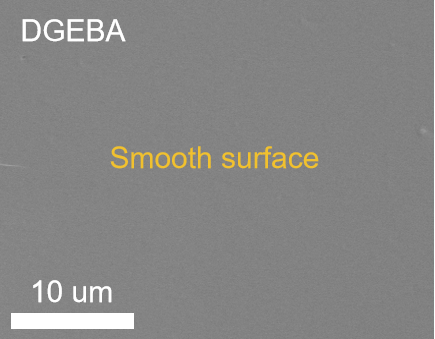


**Figure S6.** SEM image of fractured surface for DGEBA.


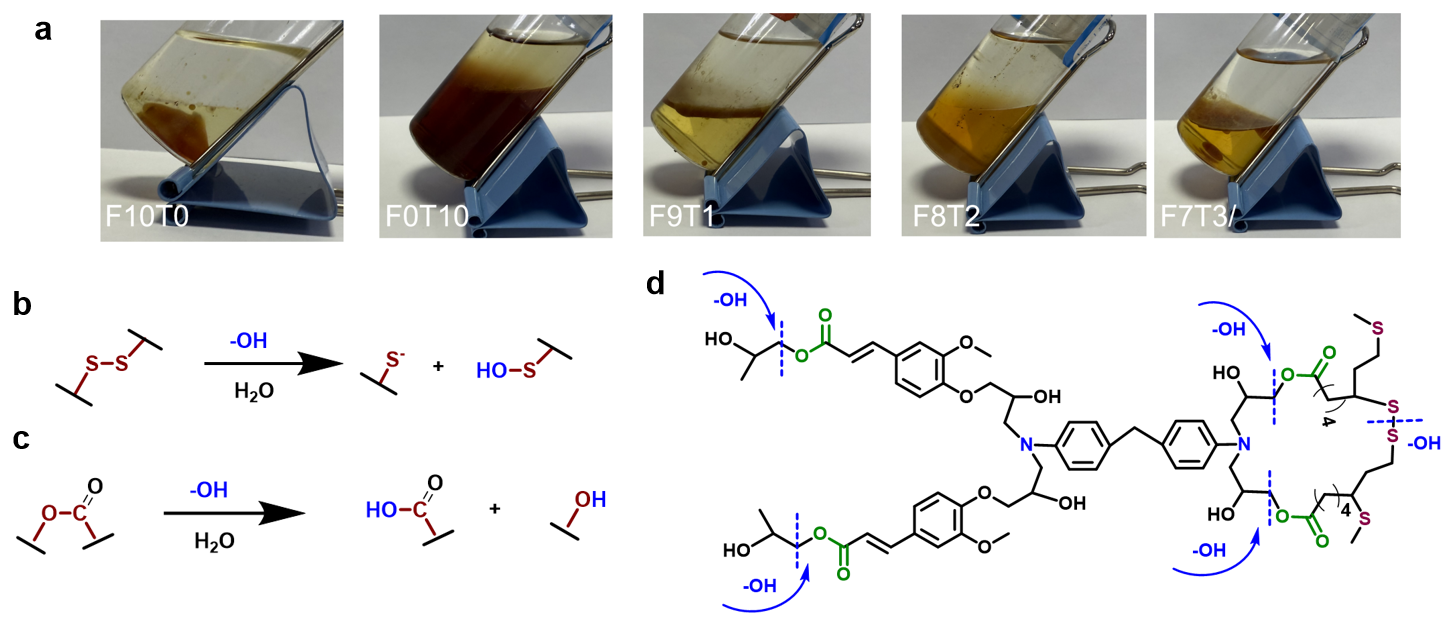


**Figure S7.** (a) Digital images of FxTy (x = 9, 8, 7; y = 1, 2, 3) degraded in THF/NaOH solution (v/v = 8:2); (b) Illustration for hydrolysis of disulfide group; (c) Illustration for hydrolysis of ester group; and (d) Proposed degradation mechanism of FxTy (x = 9, 8, 7; y = 1, 2, 3).


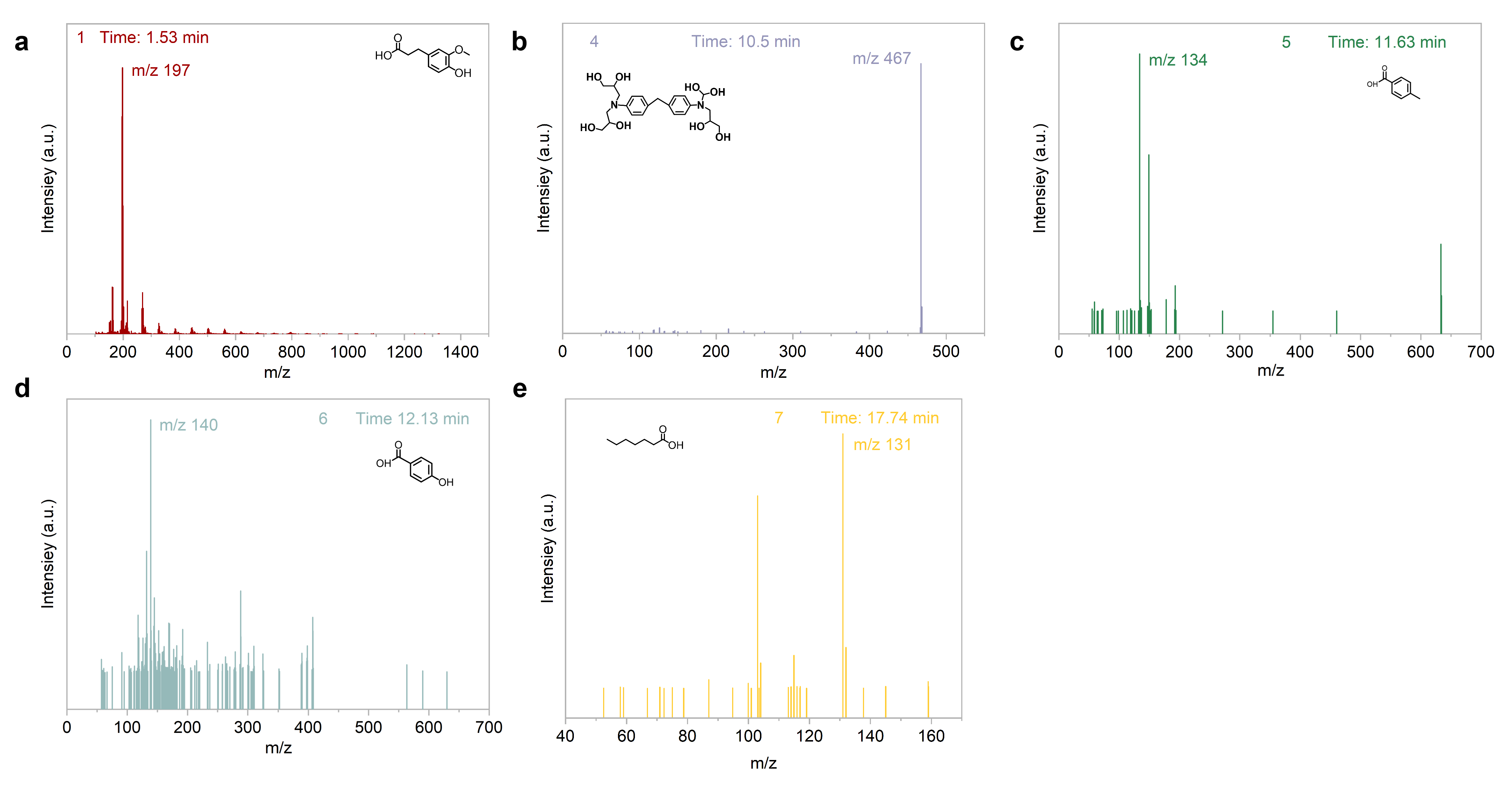
 **Figure S8.** HPLC-MS chromatogram. (a) peak 1 at 1.53 min; (b) peak 4 at 10.5 min; (c) peak 5 at 11.63 min; (d) peak 6 at 12.13 min and (e) peak 7 at 17.74 min.


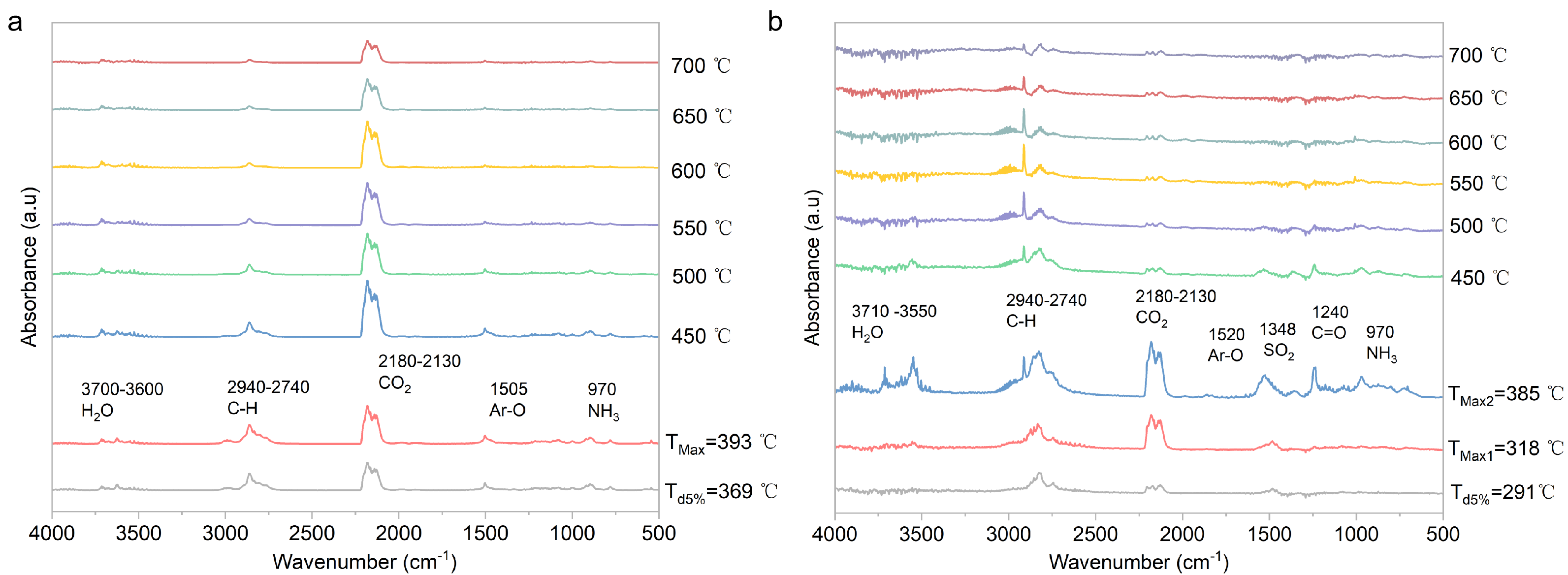


**Figure S9.** FTIR spectra of gaseous decomposition products for (a) DGEBA and (b) F9T1 samples at different temperatures.


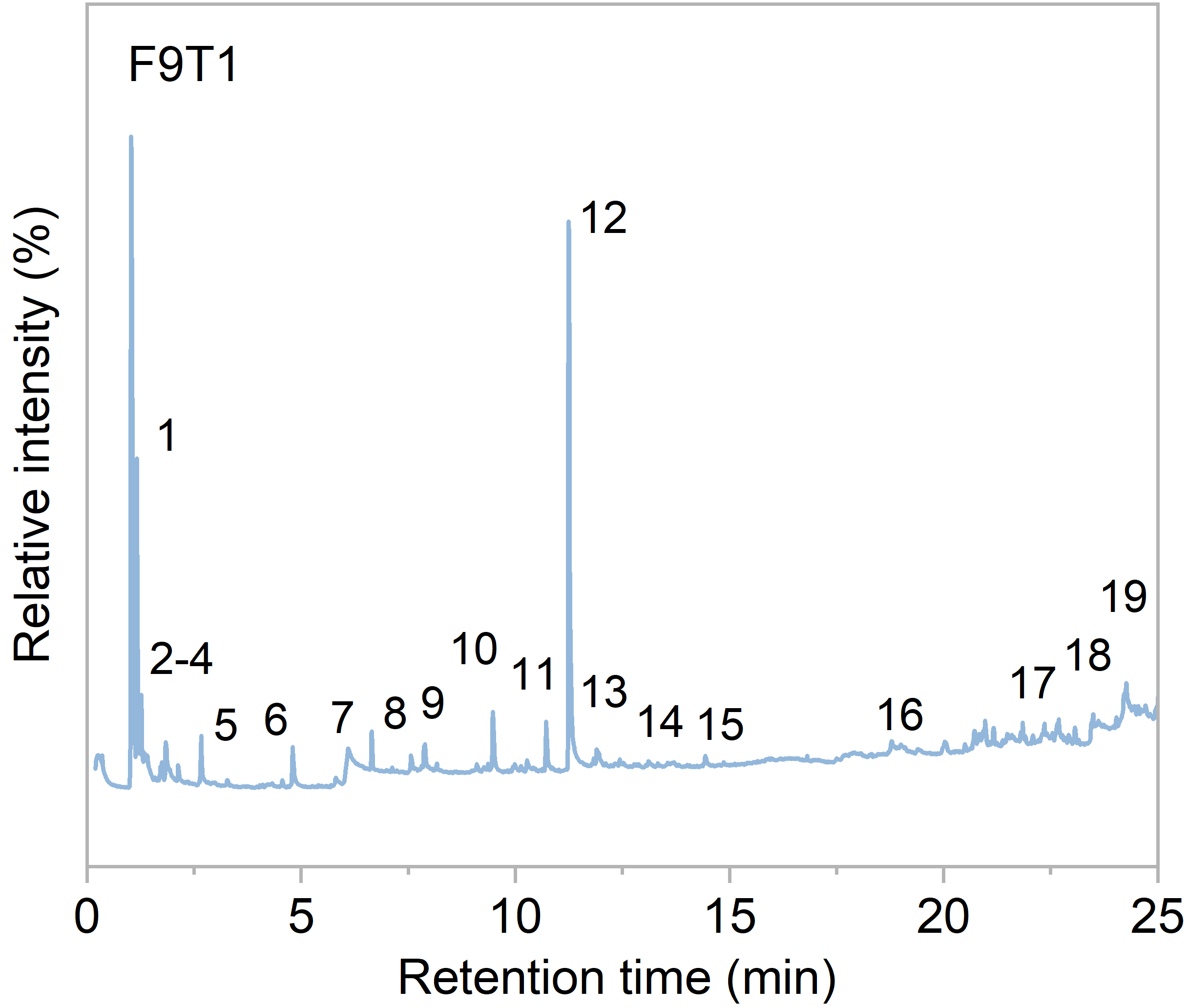


**Figure S10.** Total ion chromatogram of F9T1.

**
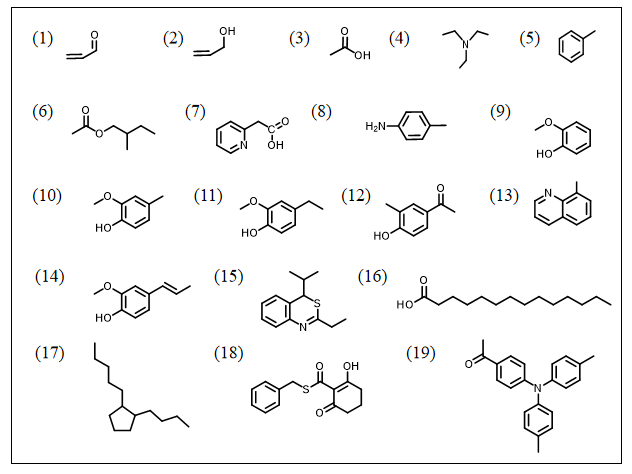
**

**Figure S11.** Possible pyrolysis fragments of F9T1 from Py-GC/MS.

**
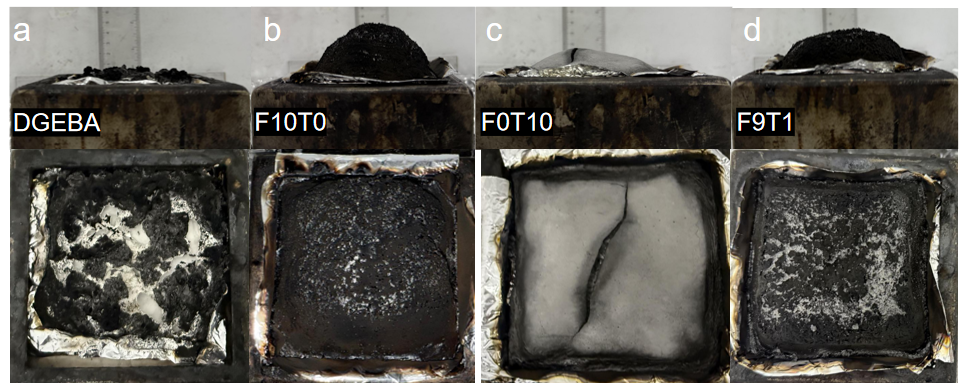
**

**Figure S12.** The digital photos of (a) DGEBA, (b) F10T0, F0T10 and (d) F9T1 char residues after CCT.


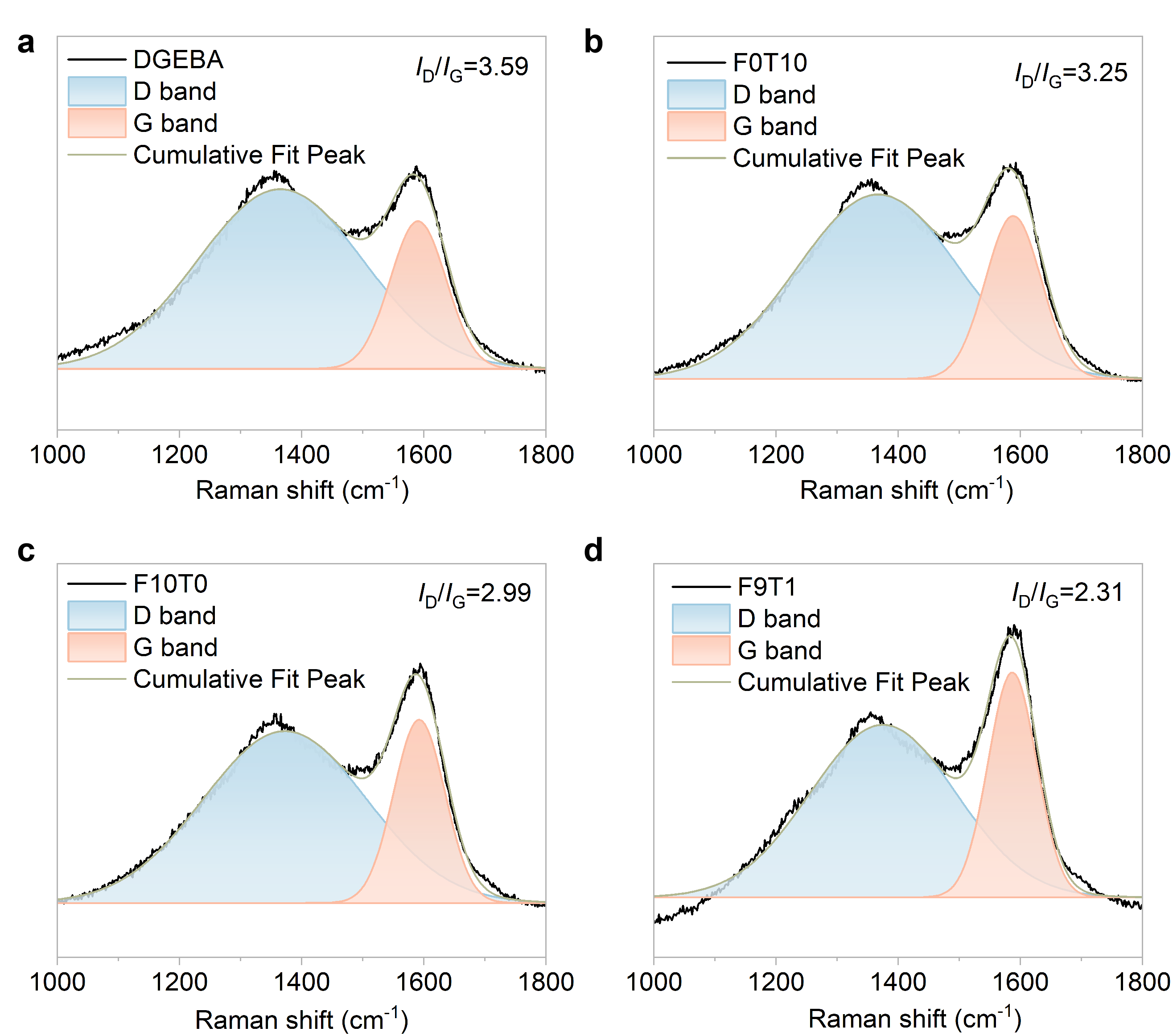


**Figure S13.** Raman spectra of (a) DGEBA, (b) F0T10, (c) F10T0 and (d) F9T1 char residues after CCT.


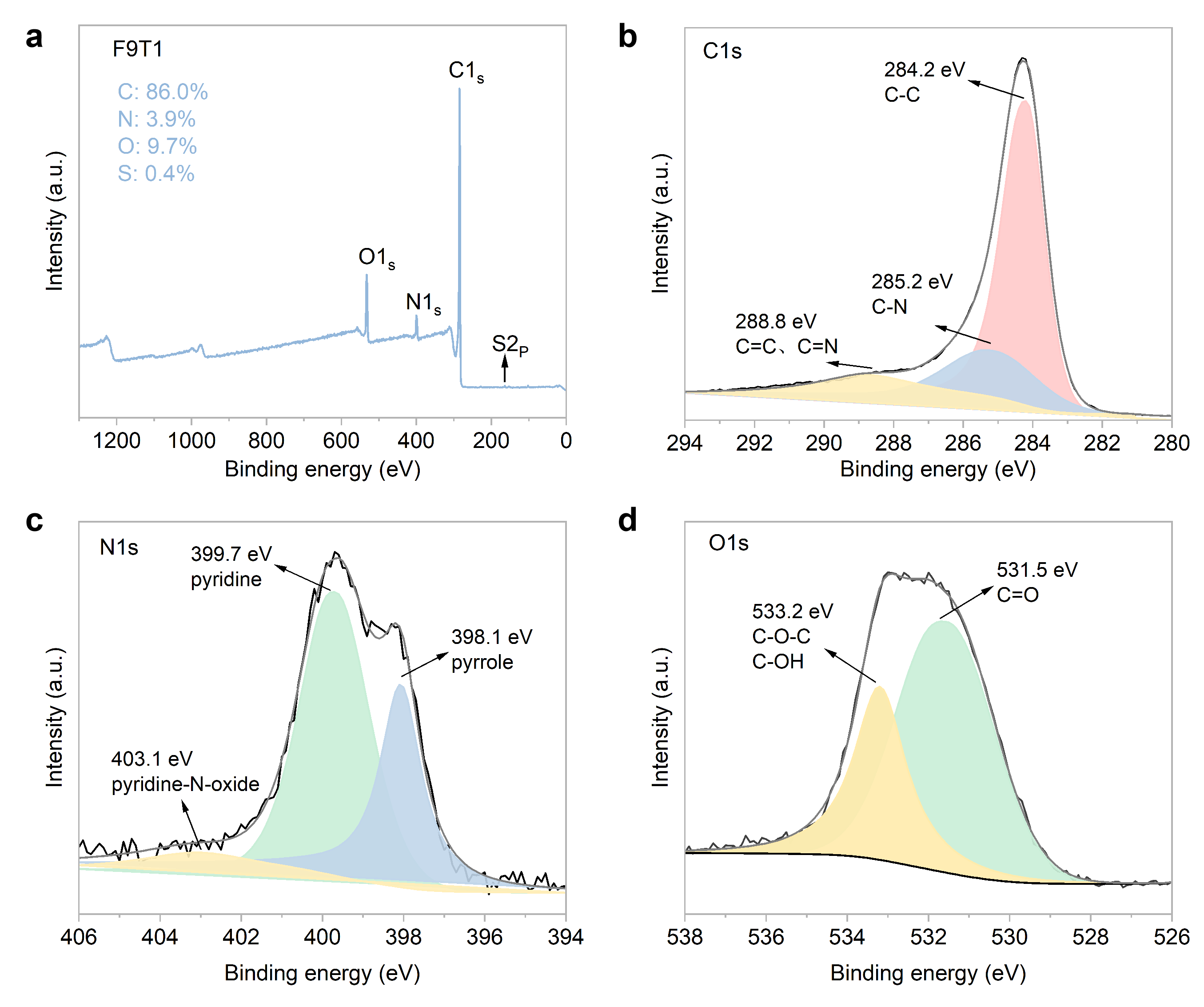


**Figure S14.** (a) Full-scan XPS spectra of F9T1 char; and High-resolution XPS (b) C1s, (c) N1s, and (d) O1s spectra of F9T1 char.


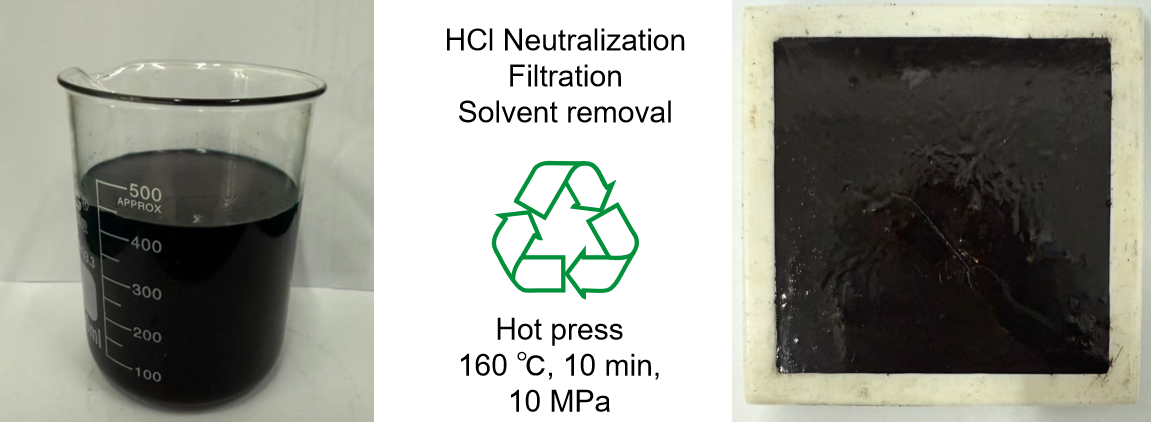


**Figure S15.** Digital images of the collected F9T1 degradation solution and the reproduced F9T1 film.


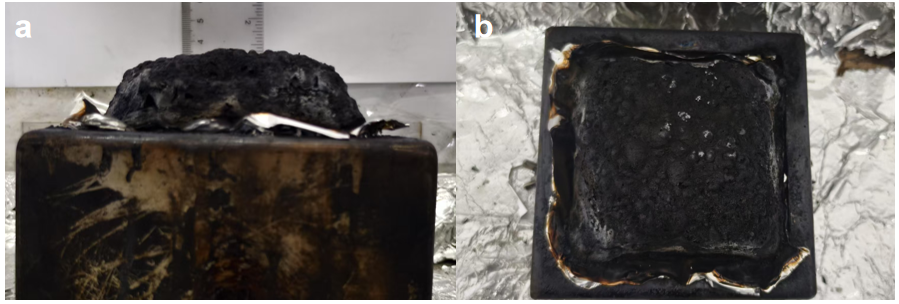


**Figure S16.** The digital photos of char for recycled F9T1 after CCT.


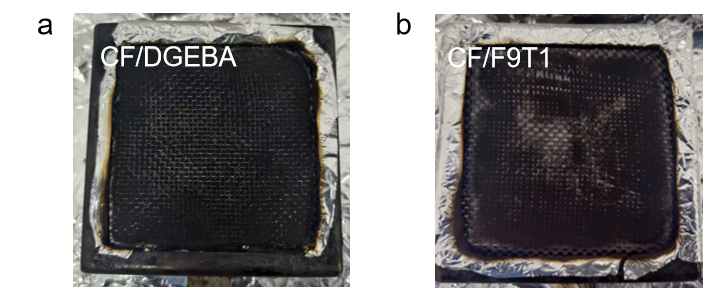


**Figure S17.** Digital photos of (a) CF/DGEBA and (b) CF/F9T1 char residues after CCT.

**Table S1.** The formulations of EP samples.

| Sample | DGEFA  (g) | DGETA  (g) | DGEBA  (g) | DDM  (g) | BCC *^a^*  (wt%) |
| --- | --- | --- | --- | --- | --- |
| DGEBA | / | / | 10 | 2.5 | / |
| F10T0 | 10 | / | / | 3 | 72.6 |
| F0T10 | / | 10 | / | 1.7 | 78.1 |
| F9T1 | 9 | 1 | / | 2.96 | 73.1 |
| F8T2 | 8 | 2 | / | 2.74 | 73.7 |
| F7T3 | 7 | 3 | / | 2.61 | 74.2 |

*^a^* Bio-based carbon content.

**Table S2.** DSC results of different epoxy systems.

| Sample | *T*_p_ (℃) | | | | *E*_a_  (kJ/mol) |
| --- | --- | --- | --- | --- | --- |
|  | 5 ℃/min | 10 ℃/min | 15 ℃/min | 20 ℃/min |  |
| F10T0 | 120.1 | 136.5 | 148.5 | 156.7 | 46.4 |
| F0T10 | 146.9 | 163.7 | 174.8 | 183.7 | 52.9 |
| F9T1 | 121.7 | 139.5 | 150.5 | 159.7 | 45.0 |
| F8T2 | 123.5 | 141.3 | 152.5 | 161.7 | 45.2 |
| F7T3 | 124.1 | 141.5 | 153.0 | 161.7 | 46.1 |

*T*_p_: Peak curing temperature; and *E*_a_: Curing activation energy calculated using the Kissinger method.

**Table S3.** Thermal stability data of EP samples in N_2_ condition.

| Sample | *T*_5%_  (°C) | *T*_max1_  (°C) | *T*_max2_  (°C) | *R*_800_  (%) |
| --- | --- | --- | --- | --- |
| DGEBA | 369 | 393 | / | 26.9 |
| F10T0 | 282 | 316 | 388 | 37.5 |
| F0T10 | 272 | 314 | / | 28.1 |
| F9T1 | 291 | 318 | 385 | 36.8 |
| F8T2 | 287 | 317 | 381 | 35.0 |
| F7T3 | 284 | 318 | 378 | 33.2 |

*T*_5%_: Temperature at 5% weight loss; *T*_max_: Temperature at maximum weight loss rate; and *R*_800_: Char yield at 800 °C.

**Table S4.** Thermal and mechanical properties of EP samples.

| Sample | *E*' at 50 °C  (MPa) | *T*_g_  (°C) | *V*_e_  (10^3^ mol/m^3^) | σ_t_  (MPa) | δ  (%) | TT  (MJ/m³) | σ_f_  (MPa) | IS  (kJ/m^2^) |
| --- | --- | --- | --- | --- | --- | --- | --- | --- |
| DGEBA | 895 | 158 | 1.00 | 56.7±3.0 | 9.9±0.6 | 3.82 | 106.5±3.2 | 5.3±0.4 |
| F10T0 | 1900 | 149 | 1.76 | 106.5±7.4 | 13.9±1.3 | 11.41 | 162.6±4.5 | 2.5±0.3 |
| F0T10 | 563 | 74 | 2.19 | 21.3±1.8 | 3.4±0.2 | 0.30 | 50.0±2.3 | 3.2±0.2 |
| F9T1 | 1540 | 153 | 2.00 | 88.5±4.6 | 11.8±0.5 | 6.74 | 137.4±1.9 | 3.4±0.3 |
| F8T2 | 1510 | 135 | 1.67 | 68.6±4.8 | 6.3±0.4 | 2.03 | 120.5±4.5 | 3.8±0.2 |
| F7T3 | 1470 | 130 | 1.64 | 44.7±4.1 | 3.7±0.3 | 0.72 | 84.1±5.9 | 3.9±0.3 |

*V*_e_: Crosslinking density; σ_t_: Tensile strength; δ: Elongation at break; σ_f_: Flexural strength; TT: Tensile toughness and IS: impact strength.

**Table S5.** Tensile strength of F9T1 before and after different aging tests.

| Sample | Tensile strength (MPa) | | |
| --- | --- | --- | --- |
|  | Original | 100 °C oven (7 d) | UV light (7 d) |
| F9T1 | 88.5±4.6 | 82.4±7.1 | 79.6±6.3 |

**Table S6.** T_g_ and mechanical properties of F9T1, previous vitrimer and bio-based EPs.

| Reference | Sample | Biobased epoxy monomer | Tensile strength (MPa) | Elongation at break (%) | Flexural strength (MPa) | T_g_  (° C) |
| --- | --- | --- | --- | --- | --- | --- |
| ^[27]^ | D_30_T_15_/EV | No | 67.4 | / | 96.7 | 121.8 |
| ^[28]^ | GHPP-0.35 | No | 72.1 | / | 86.0 | 92.0 |
| ^[29]^ | DGF/MHHPA | Yes | 84.0 | / | 96.0 | 152.0 |
| ^[29]^ | DGT/D230 | Yes | 64.0 | / | 73.0 | 91.8 |
| ^[21]^ | VNTMSi-EP | Yes | 54.5 | 13.2 | 111.0 | 94.0 |
| ^[30]^ | 0.25EIA/0.75  E51/DDM | Yes | 73.1 | 12.1 | 110.2 | 53.0 |
| ^[31]^ | BPEO/NNED | Yes | 29.3 | 9.4 | 54.9 | 29.3 |
| ^[32]^ | DGEDC/TA/PACM | No | 70.0 | / | 101.0 | 101.0 |
| ^[33]^ | DPEA-0.30 | No | 72.9 | 3.9 | 110.0 | 88 |
| ^[19]^ | BMP-EP | No | 50.5 | / | 65.9 | 162 |
| **This work** | **F9T1** | **Yes** | **88.5** | **11.8** | **137.7** | **153** |

**Table S7.** LOI and cone calorimetry data of EP samples.

| Sample | LOI  (%) | TTI  (s) | T_pHRR_  (s) | pHRR  (kW/m²) | THR  (MJ/m²) | AEHC  (MJ/kg) | RWF  (%) | TSP  (m^2^) | PSPR  (m^2^/s) | FPI  ((m^2^ s)/kW) | FGR  (kW/(m^2^ s)) |
| --- | --- | --- | --- | --- | --- | --- | --- | --- | --- | --- | --- |
| DGEBA | 25.4 | 84±3 | 102±5 | 927.1±61.9 | 80.1±0.5 | 25.8±1.0 | 11.3±2.2 | 26.5±2.0 | 0.265±0.023 | 0.091 | 9.1 |
| F10T0 | 29.9 | 48±1 | 56±4 | 530.7±83.5 | 38.6±3.2 | 16.3±1.0 | 30.8±1.4 | 13.0±1.7 | 0.198±0.001 | 0.090 | 9.5 |
| F0T10 | 27.5 | 49±3 | 61±5 | 358.1±19.7 | 46.3±1.9 | 17.6±0.3 | 19.8±1.1 | 14.2±1.2 | 0.102±0.003 | 0.137 | 5.9 |
| F9T1 | 28.7 | 56±3 | 75±9 | 503.0±45.8 | 36.4±1.4 | 15.6±0.7 | 29.5±2.1 | 12.6±0.1 | 0.202±0.013 | 0.111 | 6.7 |
| F8T2 | 28.5 | 57±3 | 71±1 | 499.1±14.1 | 35.3±0.5 | 15.0±0.1 | 29.8±0.5 | 13.2±0.2 | 0.195±0.014 | 0.114 | 7.0 |
| F7T3 | 27.7 | 55±7 | 66±5 | 412.6±30.8 | 35.0±3.2 | 15.1±0.5 | 27.8±2.1 | 13.2±0.3 | 0.152±0.004 | 0.133 | 6.3 |

TTI: Time to ignition, T_pHRR_: time to peak heat release rate, pHRR: peak heat release rate, THR: total heat release, AEHC: average effective heat of combustion, RWF: residual weight fraction, TSP: total smoke production, PSPR: peak smoke production rate, FPI: fire performance index, and FGR: fire growth index.

**Table S8.** The pHRR, THR and TSP reductions of F9T1 and previously reported epoxy resins.

| References | Sample | Phosphorus free | pHRR reduction (%) | THR reduction (%) | TSP reduction (%) |
| --- | --- | --- | --- | --- | --- |
| ^[38]^ | EP/IPD-5 | No | 33 | 25.9 | 21.1 |
| ^[2]^ | EEU-DDS | Yes | 36.3 | 31.1 | 47.9 |
| ^[39]^ | EP/9 %U-DC | No | 40.5 | 26.7 | 10 |
| ^[40]^ | EP/2K-NiPS/3DOPO | No | 44.6 | 14.4 | 37.8 |
| ^[41]^ | 15BPPDN/EP | Yes | 22.2 | 20.4 | 38.7 |
| ^[42]^ | EP/DDPS100% | No | 31.3 | 36.6 | 38.4 |
| ^[43]^ | EP10/Si-DP7 | No | 43.5 | 28.7 | 21.4 |
| ^[44]^ | EP/DDM/6.0% BDHPD | No | 37.7 | 19.8 | 11.0 |
| ^[45]^ | EP/10 wt% DOPO-DA | No | 26.1 | 32.1 | 18.5 |
| ^[19]^ | BMP-EP | No | 34.0 | 31.1 | 42.5 |
| ^[46]^ | EP/DPOA-3 | No | 25.6 | 10 | 5 |
| ^[22]^ | A_7_P_3_-D230 | No | 48.0 | 32.0 | NA |
| ^[47]^ | EP/PPCANT-5 | No | 32.6 | 22.3 | 20.1 |
| ^[48]^ | EP/PDPO-5 | No | 19.8 | 18.1 | 23.8 |
| ^[49]^ | EP/PALO-10 | No | 40.4 | 23.3 | 36.3 |
| **This work** | **F9T1** | **Yes** | **45.7** | **54.6** | **52.5** |

**Table S9.** The relative atomic content of F9T1 char determined by XPS.

| Sample | C (wt%) | N (wt%) | O (wt%) | S (wt%) |
| --- | --- | --- | --- | --- |
| F9T1 char | 86.0 | 3.9 | 9.7 | 0.4 |

**Table S10.** Tensile and interlaminar shear strength properties of CFRPs.

| Sample | Tensile strength  (MPa) | Elongation at break  (%) | Interlaminar shear strength  (MPa) |
| --- | --- | --- | --- |
| CF/DGEBA | 522±23 | 1.5±0.2 | 37.1±2.9 |
| CF/F9T1 | 621±11 | 2.7±0.1 | 44.7±2.9 |

**Table S11.** Cone calorimetry data of CFRPs.

| Sample | TTI  (s) | T_pHRR_  (s) | pHRR  (kW/m²) | THR  (MJ/m²) | AEHC  (MJ/kg) | RWF  (wt%) | TSP  (m^2^) | TSR  (m^2^/s) | FPI  ((m^2^ s)/kW) | FGR  (kW/(m^2^ s)) |
| --- | --- | --- | --- | --- | --- | --- | --- | --- | --- | --- |
| CF/DGEBA | 98±3 | 129±1 | 274.9±6.2 | 18.6±0.3 | 19.9±0.2 | 77.2±0.4 | 9.6±0.2 | 1090±19 | 0.356 | 2.1 |
| CF/F9T1 | 85±4 | 104±11 | 164.7±6.8 | 10.4±0.5 | 17.3±0.5 | 84.2±0.6 | 3.8±0.3 | 422±28 | 0.516 | 1.6 |

**Table S12.** Cone calorimetry data of F9T1 and chemically recycled F9T1 samples.

| Sample | pHRR  (kW/m²) | THR  (MJ/m²) | AEHC  (MJ/kg) | RWF  (wt%) | TSP  (m^2^) | TSR  (m^2^/s) |
| --- | --- | --- | --- | --- | --- | --- |
| F9T1 | 503.0±45.8 | 36.4±1.4 | 15.6±0.7 | 29.5±2.1 | 12.6±0.1 | 1424±17 |
| Recycled F9T1 | 293.1±59.3 | 27.4±3.8 | 18.8±0.9 | 41.0±2.6 | 5.4±0.2 | 605±24 |
